# Mollusk-Derived Peptides at the Food-Pharma Interface: Bioactivities, Preclinical Evidence, and Development Prospects

**DOI:** 10.3390/molecules31183186

**Published:** 2026-09-10

**Authors:** Muhammad Imran, Yunyan Li, Muhammad Asif, Muhammad Azam, Kainat Aleem, Zhenyan Jiang, Muhammad Adil, Yanglei Yi

**Affiliations:** 1 College of Food Science and Engineering, Northwest A&F University, Yangling 712100, China; imranbhatti973@gmail.com (M.I.); yunyan_li2026@nwafu.edu.cn (Y.L.); muhammadazamfst@gmail.com (M.A.); 2 National Institute of Food Science and Technology, University of Agriculture Faisalabad, Faisalabad 38000, Pakistan; asifrajab3251@gmail.com; 3 College of Forestry, Northwest A&F University, Yangling 712100, China; kainataleem2322@nwafu.edu.cn; 4 School of Pharmaceutical Sciences, Jilin University, Changchun 130021, China; 5 School of Food Science and Engineering, South China University of Technology, Guangzhou 510641, China

**Keywords:** mollusk-derived peptides, enzymatic hydrolysis, translational challenges, food-pharma, peptide bioactivities

## Abstract

Mollusk-derived peptides are an expanding class of marine bioactive molecules that link food-protein utilization with pharmaceutical peptide discovery. They are derived from taxonomically and biologically diverse sources such as edible gastropods, bivalves, cephalopods and highly modified conopeptides from cone snails. However, there is a lack of integrated understanding of sources, structures and functions of these peptides and their potential applications in food science, pharmacology, and venom research. In this review, the current knowledge related to mollusk-derived peptides from a food–pharma perspective is summarized, including taxonomic sources, tissue substrates, enzymatic hydrolysis, purification, sequence identification, reported bioactivities and structure–function relationships. The peptides derived from food are primarily short linear peptides from muscle, mantle, viscera, collagen, gelatin and processing by-products. These peptides have mainly been investigated for antioxidant, Angiotensin-I-converting enzyme inhibitory, anti-inflammatory, mineral-binding, gut-health, and metabolic activities. However, the biological functions of endogenous peptides and peptides from cone snails are often associated with cysteine-rich scaffolds and disulfide connectivity and post-translational modifications that confer antimicrobial activity, neuromodulation, and receptor selectivity. For these systems, the activity depends not on any single parameter but on the peptide length, amino acid sequence, charge distribution, hydrophobicity, amphipathicity, secondary structure and chemical modifications. Further progress requires improved sequence-level validation, mechanism-driven bioassays, in vivo and clinical evidence, safety assessment, and feasible purification and delivery strategies for mollusk peptides into functional foods, nutraceuticals and peptide-based therapeutics.

## 1. Introduction

Marine organisms represent a productive source of bioactive peptide discovery, owing to the unusual selective pressures of the marine environment, which favor the evolution of structurally diverse and functionally potent proteins not typically found in terrestrial sources [1]. Enzymatic hydrolysis of these marine proteins can release short peptides with antioxidant, antihypertensive, antimicrobial, anti-inflammatory and anticancer activities, positioning marine-derived peptides as candidates for both functional food ingredients and pharmaceutical leads [2,3]. Within this broader marine peptide landscape, the phylum Mollusca, the second-largest animal phylum by species number, represents a particularly rich and taxonomically diverse reservoir of bioactive molecules, spanning gastropods, bivalves and cephalopods, each contributing distinct tissue types, protein substrates and ecological adaptations to the pool of candidate peptides [4].

Two broadly distinct lines of evidence currently support interest in mollusk peptides. On one side, edible bivalves and gastropods such as oysters, mussels, clams, scallops and abalone yield short, linear peptides through enzymatic hydrolysis of muscle, mantle, viscera, collagen, gelatin and processing by-products; these food-derived peptides have been associated with antioxidant, angiotensin-i-converting enzyme inhibitory, anti-inflammatory, mineral-binding, gut-health and metabolic activities and are increasingly being explored for functional food and nutraceutical applications [5]. On the other side, cone snails (Conus spp.) represent a pharmacologically specialized gastropod lineage whose venom gland precursors are processed into conopeptides or conotoxins, many of which are stabilized by disulfide bonding and further diversified by extensive post-translational modifications; these venom peptides act with high potency and selectivity on ion channels and neurotransmitter receptors, and one such conopeptide, ziconotide (a synthetic version of ω-conotoxin MVIIA from Conus magus), was approved by the Food and Drug Administration (FDA) as an intrathecal analgesic for severe chronic pain, remaining one of the clearest examples of a marine peptide translated into approved clinical use [6]. The chemical diversity of this venom system is considerable, as a single Conus species may produce more than one thousand distinct conopeptides. Bibliometric evidence also indicates sustained international growth in conotoxin research, particularly in structure–activity relationships and medicinal chemistry [7,8].

A recent review comprehensively summarized conotoxins that selectively target voltage-gated sodium (NaV) channels, a major class of ion channels implicated in pain signaling, cardiac excitability, and neuronal function. This review systematically summarized the diverse conotoxin subfamilies acting on NaV channels, including their distinct binding sites, subtype selectivity across the nine known human NaV isoforms, and structure–activity relationships. It also highlighted their potential as pharmacological tools and therapeutic leads for conditions such as chronic pain and arrhythmia. The continued characterization of NaV-targeting conotoxins underscores the pharmacological richness of the *Conus* venom system and its ongoing relevance to ion channel drug discovery [9].

Despite this expanding literature, mollusk-derived peptides have generally been reviewed either from a food-science and nutraceutical perspective, emphasizing hydrolysis, purification and functional bioactivity of edible tissues, or from a venom-pharmacology perspective, emphasizing conopeptide structure, receptor selectivity and drug development [10]. Reviews addressing molluscan bioactive compounds more broadly, including nutraceutical and secondary-metabolite surveys covering terpenoids, sterols, and related classes [4,11], have provided valuable overviews of mollusk-derived bioactivity but have not offered a peptide-specific framework connecting tissue source, hydrolysis and purification strategy, sequence-level identification, and structure–function relationships across both the food-derived and venom-derived branches of molluscan peptide biology.

**Figure 1:**
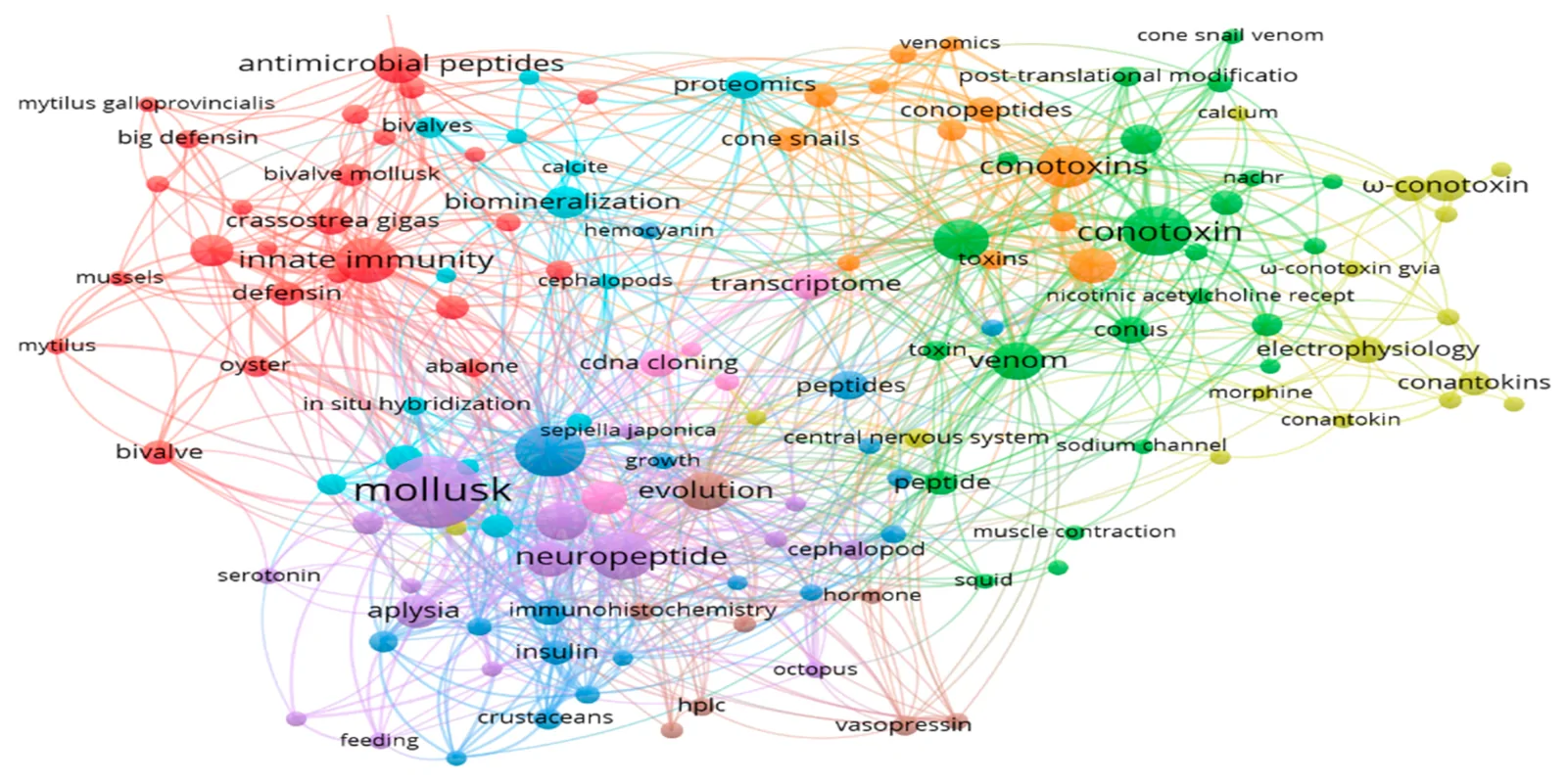
Keyword co-occurrence network analysis of mollusk-derived peptides at the food–pharma interface. Note: The VOSviewer (1.6.21) overlay map shows major research clusters related to mollusk sources, peptide structures, antimicrobial and neuroactive bioactivities, conotoxins, omics-based discovery and translational potential.

Likewise, reviews of shellfish peptides tend to remain within the food and nutraceutical domain, addressing extraction, characterization and commercial potential without extending the discussion to the post-translationally modified, receptor-targeted peptides that define cone snail pharmacology [5]. This separation has left a clear gap: no single framework currently integrates together the taxonomic sources, tissue substrates, hydrolysis and purification pipelines, reported bioactivities and structure–function principles of mollusk peptides in a way that spans both the food-pharma continuum and the more specialized venom pharmacology of conopeptides. Figure 1 presents the keyword co-occurrence network analysis of research on mollusk-derived peptides at the food-pharma interface. The map highlights the evolution of scientific attention toward major peptide-related themes, including mollusk sources, antimicrobial peptides, conotoxins, neuropeptides, proteomics, transcriptomics, venomics, innate immunity, biomineralization, and pharmacological bioactivities. Larger nodes represent frequently occurring keywords, while the links indicate the strength of co-occurrence between terms.

Bridging these research areas is scientifically and translationally important. Edible mollusks and their processing by-products represent an underused, sustainable source of peptides for functional foods and nutraceuticals, but their development is constrained by inconsistent purification pipelines, limited in vivo and clinical validation, and unresolved safety and allergenicity concerns [5]. From a pharmaceutical standpoint, the post-translationally elaborated peptides of cone snail venom continue to yield leads for neurological, analgesic and other indications, yet their pharmacological logic, structure–function principles and delivery challenges are rarely discussed alongside the biologically distinct, but chemically related, peptides derived from edible mollusk tissues [6]. Integrating these fields permits peptide length, charge distribution, hydrophobicity, amphipathicity, secondary structure, and chemical modification to be considered within a shared structure–function framework.

Within this scope, we summarize current knowledge of mollusk-derived peptides from an integrated food-pharmaceutical perspective. The search strategy and eligibility criteria are provided in the Supplementary Methods. Beginning with their taxonomic origins and tissue sources across gastropods, bivalves, and cephalopods, the review then traces the enzymatic hydrolysis, purification, and sequence-identification workflows used to transform molluscan biomass into validated bioactive candidates. This provides a foundation for examining their major reported bioactivities, including antihypertensive, anti-inflammatory, antioxidant, anticancer, mineral-binding, anti-fatigue, metabolic, and gut health-promoting effects. Within this broader landscape, particular attention is given to the extensive post-translational modifications of venom-derived conopeptides, which distinguish them from the predominantly unmodified short peptides obtained from food sources. The discussion subsequently considers the emerging applications of mollusk-derived peptides and collagen in food and pharmaceutical development before addressing the principal barriers to translation, including production scalability, purification costs, stability, bioavailability, safety assessment, and regulatory approval.

## 2. Taxonomic Overview of Mollusk-Derived Peptides

**Figure 2:**
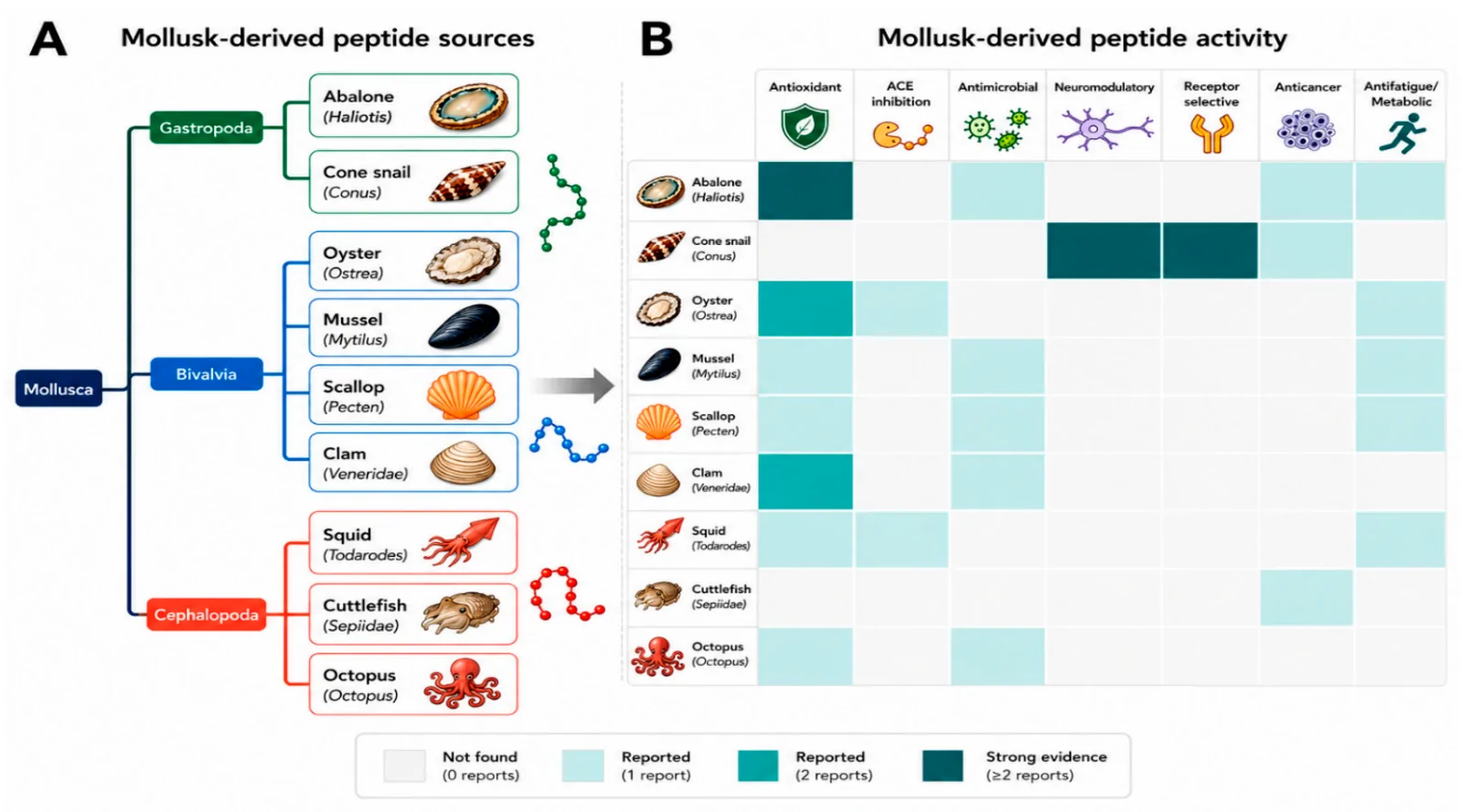
Taxonomic distribution and reported bioactivities of mollusk-derived peptides. (**A**) Classification of Mollusca (Gastropod, Bivalvia, Cephalopoda) with representative peptide structures. (**B**) Heatmap of reported bioactivities across nine species (2020–2026).

Mollusks can be classified into three major peptide-producing classes: Gastropod, Bivalvia, and Cephalopoda. Figure 2 summarizes the three molluscan classes and the bioactivities studied between 2020 and 2026. In the Gastropod class, *Abalone* (*Haliotis* spp.) is a food-protein source in which peptides with antioxidant and other functional properties can be obtained by enzymatic digestion of muscle, viscera and collagen-rich tissues. Recent work on *Haliotis discus hannai* identified abalone-derived antioxidant peptide candidates, including DEDEDEDK, through peptide-library screening and activity validation [12]. Another study identified *Crassostrea rivularis* oyster-derived antioxidant peptide candidates, including AWVDY, MSFRFY, EPLRY, RKPPWPP, and YAKRCFR, through membrane separation and UPLC-QTOF-MS/MS sequencing, with the most potent peptide (YAKRCFR) validated for protection against H_2_O_2_- induced oxidative damage in Caco-2 cells [13]. In contrast, cone snails (*Conus* spp.) are a pharmacologically specialized gastropod group. Their venom gland precursor proteins are processed to conopeptides or conotoxins, many of which contain disulfide framework and post-translational modifications that yield high-affinity modulation of neurotransmitter receptors and ion channels [14,15].

Bivalvia, including oysters (*Crassostrea* spp.), mussels (*Mytilus* spp.), scallops (*Patinopecten* spp.) and clams (*Ruditapes philippinarum*), provide both endogenous immune peptide and food-protein hydrolysates. Mussels (*Mytilus galloprovincialis*) produce cysteine-rich antimicrobial peptides such as myticin, mytilin and defensins in haemocytes and plasma, whereas oysters and clams have yielded ACE-inhibitory peptides after fermentation or enzymatic hydrolysis, including peptides from fermented oyster sauce and the *Cyclina sinensis* (commonly known as Chinese venus, black clam, iron clam, and Korean cyclina clam) pentapeptide WPMGF [16]. In Cephalopoda, squid, cuttlefish and octopus peptides are derived primarily from muscle protein, collagen, gelatin, skin, tunic and processing by-products. Squid-gelatin and cuttlefish-muscle hydrolysates have been associated with antioxidant and ACE-inhibitory activities [17,18].

Within Cephalopoda, squid represent the most extensively studied group. Skin gelatin hydrolysates from the jumbo squid (*Dosidicus gigas*), produced by tryptic or Alcalase digestion, have yielded well-characterized antioxidant peptides, including Phe-Asp-Ser-Gly-Pro-Ala-Gly-Val-Leu and Asn-Gly-Pro-Leu-Gln-Ala-Gly-Gln-Pro-Gly-Glu-Arg. These peptides show strong lipid-peroxidation inhibition exceeding that of α-tocopherol and effective scavenging of hydroxyl and carbon-centered radicals [17]. In the Japanese flying squid (*Todarodes pacificus*), skin-derived hydrolysates have similarly been screened for angiotensin-converting enzyme (ACE)-inhibitory peptides, with low-molecular-weight fractions showing high ACE-inhibitory potency attributable to their hydrophobic amino acid content, alongside good thermal and pH stability [19].

These peptide resources also differ by their mode of production, which helps predict their application. The first source type is endogenous defense and venom peptides including mussel defensins, mytilins and myticins as well as conopeptides. These peptides are gene-encoded, proteolytically processed, and often stabilized by disulfide bonds, amidation and other post-translational modifications for antimicrobial, antiviral and receptor-selective drug discovery usage [20]. The second source comprises food-derived peptides produced enzymatically by using enzymes such as pepsin, trypsin, alcalase and simulated gastrointestinal digestion from mollusk muscle, collagen, gelatin, viscera, or processing by-products. These peptides are usually shorter and more heterogeneous than immune and venom peptides. These peptides have antioxidant, ACE-inhibitory, anti-inflammatory and mineral binding activities and are well suited to functional-food development [17]. Processing-derived peptides are the third source type, such as fermented oyster sauce by endogenous and microbial proteases which release bioactive peptides during food processing. It is necessary to consider endogenous mollusk peptides as immune or pharmacological molecules while hydrolysate and processing peptides are more naturally considered by means of digestibility, bio accessibility, sensory acceptability, scalability, and food safety parameters.

## 3. From Mollusk Biomass to Bioactive Peptides

Marine mollusks represent more than a conventional seafood protein source. They are rich in structurally diverse proteins, which upon processing can be transformed into bioactive peptides that may be useful in functional foods, nutraceuticals, and the pharmaceutical industry. The transition from mollusk biomass involves selection of species and tissue, characterization of protein substrate, peptide release, sequence identification, stability, delivery assessment, safety and regulatory evaluation. However, mollusk tissues like mantle, adductor muscle and foot vary in protein type by function and may affect the peptide profile following the enzymatic hydrolysis or digestion. Different patterns of amino acids and distinct molecular weights, charge distribution and biological activity of the peptides may result from a tissue rich in proteins related to structure, contraction, or mineralization. Therefore, tissue origin should be considered a determinant of peptide diversity and downstream function.

### 3.1. Mantle Tissue as a Protein Substrate for Peptide Generation

The mantle of a mollusk is a multi-purpose tissue that is common to all mollusks. It is responsible for lining the inner surface of the shell, containing the visceral mass, and forming part of the mantle cavity and is involved in respiration, excretion, reproduction, and formation of the shell. Its function in shell secretion and repair makes it particularly interesting regarding peptide research, as the proteins of the mantle participate in biomineralization, matrix formation, and environmental adaptation [21]. In certain species, the mantle is involved in special activities such as sensory appendage development in scallops and light-producing organs in deep-sea cephalopods [22,23]. The variety of biological functions suggests that the mantle tissue may contain proteins with structural, adhesive, regulatory and defense properties.

The mantle is a protein-rich, functionally specialized substrate for peptide discovery. Protein levels vary between species and can be affected by diet, habitat, season, reproductive stage, and environmental conditions. In commercially edible bivalve mantle tissues, such as those of *Anadara brougtonii* and *Mactra chinensis*, substantial protein content has been found, although the level differs among species [24]. Based on these observations, it can be inferred that peptides originating from the mantle preparations should not be considered as a general mollusk group but should be species specific, depending on the tissue state and processing conditions.

### 3.2. Adductor Muscle as a Contractile Protein Reservoir

One of the most significant protein-rich tissues in bivalve mollusks (e.g., mussels, scallops, oysters, and clams) is the adductor muscle. Its main purpose is to keep the two shell valves closed, safeguarding the animal against predators, and also to control and regulate water flow during feeding and respiration. Bivalves can have one or two adductor muscles. The tissue is specialized for speed of contraction and for shell closure over a long period, and the size and shape of the tissue are dependent upon the species. Scallops also use their adductor muscle to move, repeating the action by ‘clapping’ the shell [25]. Muscle proteins are important precursors of bioactive peptides, and due to the importance of the adductor muscle in its contractile function, the muscle proteins of this species dominate. Studies on the nutritional profile of pearl oysters such as *Pinctada maxima* and *Pinctada margaritifera* have shown that the adductor muscle has a high protein content while its lipid and cholesterol contents are low [26]. In *Anadara brougtonii* and *Mactra chinensis*, the adductor protein content was also found to be different depending on the species [24]. The adductor muscle of pen shell has also been found to contain a very high proportion of protein, with comparatively low fat and ash contents [27]. Overall, the results indicate that the adductor muscle is a promising source of peptides, especially when protein yield and minimizing lipid interference are desired. However, high protein content alone does not ensure translational value. The method for peptide release has to be optimized to produce consistent peptide profiles; the released fractions then have to be purified and characterized at the sequence level.

### 3.3. Foot Muscle and Other Underused Tissues in Peptide Discovery

The mollusk foot is used for attachment, burrowing, crawling, and other types of locomotion, and it is muscular. It exists in numerous groups of mollusks and has been of interest due to biomechanical features, protein composition, and developmental and neurobiological applications [28]. Nutritional analyses further indicate that the foot is also a protein-rich tissue. For example, the *Anodonta anatine* foot showed higher protein content as compared to other tissues such as the mantle and gills [29]. The proximate analysis of *Donax variabilis* also showed high content of protein in foot muscle with minor constituents of carbohydrates, lipids, and fatty acids [30]. The foot muscle offers special interest for translational review since it is less studied than the edible adductor muscle and might provide different peptide precursors. Its roles in locomotion, adhesion, and mechanical stress, etc., may favor proteins with structural properties distinct from those of mantle or adductor-muscle proteins. This difference may affect peptide hydrophobicity, charge, and resistance to digestion. Furthermore, foot tissue and other processing by-products could serve as the source of sustainable peptide production through the utilization of underused biomass to yield valuable products.

### 3.4. Converting Tissue Proteins into Validated Bioactive Candidates

Mollusks provide protein substrates from soft tissues, adductor muscle, viscera, mantle, and processing by-products, but most of these matrices contain varying amounts of salts and minerals, glycogen, connective proteins, shell-matrix-associated proteins, and marine contaminants. These tissue characteristics can influence enzyme accessibility, peptide release, downstream purification and safety assessment. Therefore, the value of a mollusk peptide is determined not only by its reported bioactivity, but also by the tissue source, hydrolysis strategy, sequence confirmation and evidence that the peptide remains active under physiologically relevant conditions [31,32]. Preparation strategies and characteristics of mollusk peptides are shown in Table 1.

Enzymatic hydrolysis remains the main route for producing mollusk-derived peptides because it allows controlled cleavage of tissue proteins and can be adapted to different mollusk matrices. Studies on mussel, oyster, clam, and abalone proteins commonly use proteases such as alcalase, pepsin, papain, neutrase, flavourzyme, or combined enzyme systems to generate low-molecular-weight peptide fractions [40]. However, the key issue is not simply which enzyme gives the highest degree of hydrolysis. For mollusk peptides, hydrolysis conditions have to be optimized based on target activity and type of tissue. For instance, enriched hydrolysates of oyster or mussel with small peptides tend to demonstrate greater antioxidant or ACE inhibitory effects, while zinc-binding or mineral-chelating peptides might need preservation of acidic, basic, or histidine-rich residues [41,42]. Pretreatments such as ultrasound-assisted hydrolysis and microbial fermentation can be used to improve protein breakdown and peptide liberation, but they should not be considered as processing innovations unless their application clearly leads to a better yield of mollusk peptides, sequence diversity, sensory characteristics and/or biological activity [38]. After hydrolysis, purification should narrow the complex hydrolysate into fractions that can be defined as peptide structures. Many active mollusk peptides occur in low-molecular-weight fractions, often below 1, 3, or 5 kDa. Ultrafiltration is therefore commonly used as the first enrichment step. This trend has been observed for hydrolysates of oyster, mussel, clam, and abalone; smaller hydrolysates may also exhibit greater antioxidant, ACE-inhibitory, or functional activities [43,44].

However, molecular mass is not a good indicator of activity. Further chromatographic separation, such as gel filtration, ion-exchange chromatography, and RP-HPLC are required to enrich peptides based on size, charge, and hydrophobicity. For the mollusk hydrolysates, they are particularly important when there are salts, free amino acids, minerals, and non-peptide components that may affect assays of activity. A hydrolysate-derived candidate becomes more credible after sequence identification and independent activity revalidation. The sequences of peptides are identified by LC-MS/MS, MALDI-MS, and ESI-MS/MS. Various mollusk studies have followed these methods for identification of antioxidant peptides from *Crassostrea madrasensis*, bioactive peptide fractions from *Perna canaliculus*, antioxidant peptides from abalone viscera and antimicrobial peptides from *Nerita versicolor* and *Pomacea poeyana* [45].

The final validation step should connect peptide structure with biological relevance. Properties such as chain length, hydrophobicity, charge distribution, aromatic or sulfur-containing residues, amphipathicity, and metal-binding capacity collectively influence antioxidant, ACE-inhibitory, antimicrobial, zinc-binding, and cell-protective activities. However, most mollusk-derived peptides remain supported primarily by in vitro evidence. Translation into functional foods, nutraceuticals, or pharmaceutical candidates therefore requires evaluation of gastrointestinal stability, intestinal absorption, dose–response relationships, cytotoxicity, allergenicity, and contaminant exposure. For food-directed applications, allergenicity should not be inferred solely from hydrolysis or low molecular weight. Risk-based testing should quantify residual allergenic proteins and assess IgE binding using sera from clinically characterized allergic individuals, supplemented by functional assays where appropriate. Because mollusks can bioaccumulate environmental contaminants, representative raw materials and finished-product batches should also undergo validated screening, such as inductively coupled plasma mass spectrometry, for lead, cadmium, mercury, and arsenic, including inorganic arsenic speciation where relevant.

## 4. Bioactivities Across the Food–Pharmaceutical Spectrum

**Figure 3:**
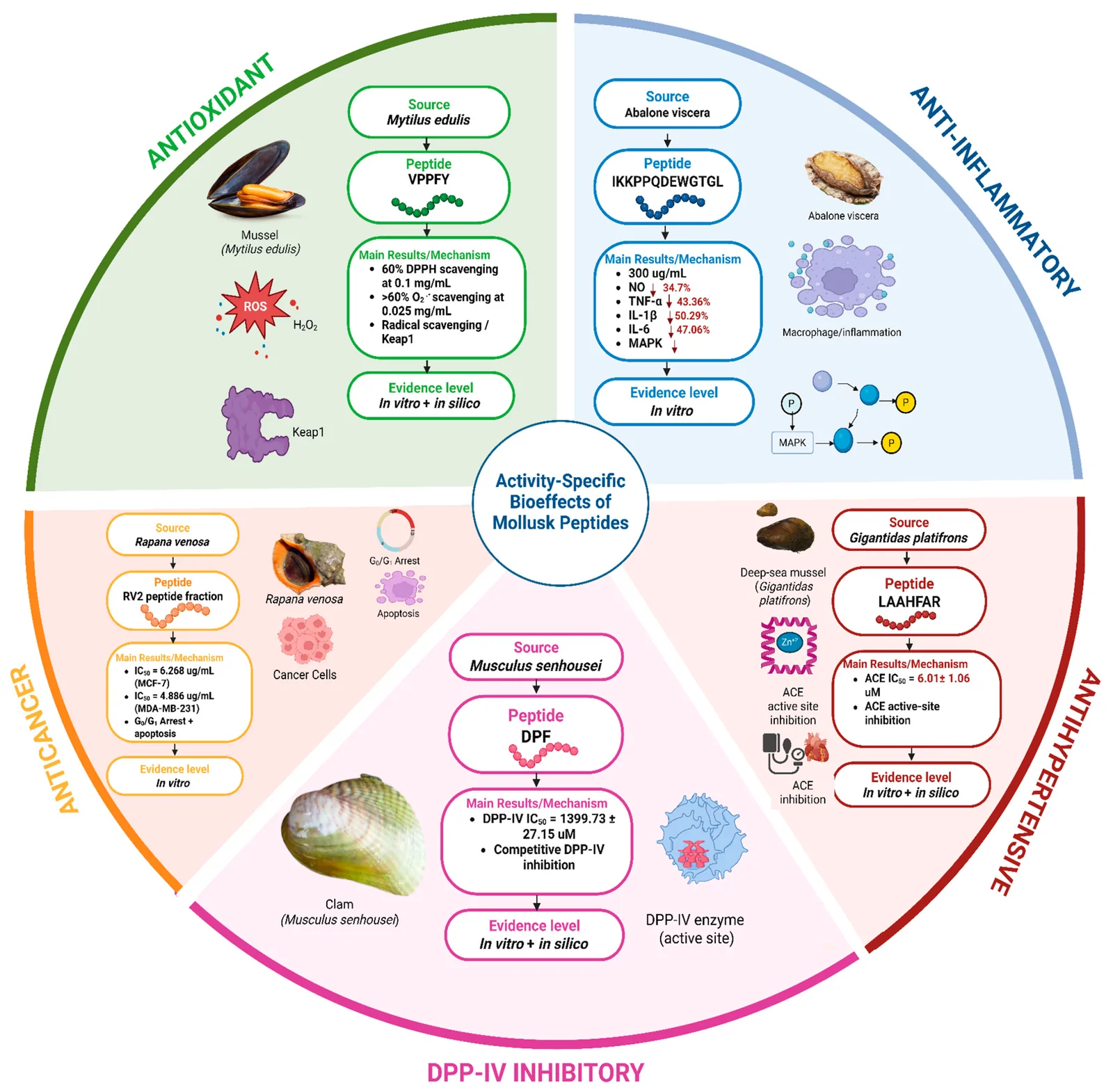
Activity-specific bioeffects of representative mollusk-derived peptides, highlighting their quantitative effects, and corresponding evidence levels.

Mollusk-derived peptides have been reported to exhibit diverse biological activities, including antioxidant, antidiabetic, antihypertensive, anti-inflammatory, and anticancer effects. These activities involve mechanisms such as radical scavenging, enzyme inhibition, modulation of cellular signaling, and protection against cellular stress. Figure 3 provides a category-level overview of activities reported for different peptide sequences, hydrolysates, mollusk species, and experimental studies. The tested concentrations, potency measures, experimental models, and evidence levels vary substantially among studies. Sequence-specific activities and their corresponding evidence levels are summarized in Table 2.

Gastrointestinal stability and intestinal bioavailability remain major limitations in translating in vitro peptide activity into oral efficacy. Among the sequence-resolved peptides summarized in Table 2, WPMGF has been evaluated under simulated gastrointestinal conditions and retained ACE-inhibitory activity [15]. AEYLCEAC was identified following simulated gastrointestinal digestion of an oyster hydrolysate and subsequently evaluated in hypertensive rats [55]; however, these findings do not establish the intestinal absorption or systemic bioavailability of the intact peptide. For most other peptides, resistance to gastrointestinal and brush-border peptidases, transepithelial transport, plasma stability, and human bioavailability have not been reported. Their in vitro potency should therefore be interpreted as preliminary screening evidence rather than evidence of oral efficacy.

### 4.1. Antihypertensive Activity via ACE Inhibition

Angiotensin-converting enzyme-I (ACE-I) is responsible for the metabolism of bradykinin, and it catalyzes the formation of angiotensin II, a potent vasopressor, which regulates blood pressure [60]. ACE inhibitors reduce angiotensin II formation and regulate the bioavailability of bradykinin. However, these drugs have several side effects such as renal impairment, angioneurotic edema, loss of taste and chronic cough [61]. So, it became crucial to identify food-derived ACE inhibitors that may offer acceptable tolerability. Various ACE inhibitory peptides have been found in different natural sources, such as mushroom, milk, cheese, soybean, walnut, and others. In marine sources, anti-hypertension peptides have been found in mussels, fish and algae [62]. The AHTPDB database can be used to get information about molecular weight, length, IC_50_, structure and purification method for anti-hypertension peptides. Suo et al. [52] isolated seventeen angiotensin converting enzyme inhibitory peptides from blue mussel (*Mytilus edulis*) and identified YEGDP, WF, NFRPQ, GPE, SWISS, SVEWK, FKWH, MFR, MFV, FV, KP, QP, QVK, IK, MLKV, YKV, and IRK. Among these peptides, YEGDP showed the strongest ACE inhibition (IC_50_ = 0.19 mg mL^−1^), followed by SWISS, WF, and IK (0.32, 0.40, and 0.77 mg mL^−1^, respectively). Docking attributed this activity to electrostatic, hydrophobic, and hydrogen-bond interactions with ACE. Although cross-study comparisons are assay-dependent, YEGDP remains several orders of magnitude less potent than lisinopril and is not currently a competitive pharmaceutical ACE inhibitor. Its immediate value is as a food-derived nutraceutical candidate, pending validation of its gastrointestinal stability, in vivo efficacy, safety, and clinical benefit.

Cudennec et al. [63] evaluated the antihypertensive potential of land snail by-product hydrolysate (SBH) and performed in vitro and in vivo studies. The ACE inhibitory activity of SBH was characterized through an IC_50_ value of 23 µg·mL^−1^ which was not affected by in vitro digestion. Snail-by-product hydrolysate increased the Caco-2 intestinal cell metabolic activity and did not show any toxicity on Wistar rats. SBH partial purification produced an active fraction, which was characterized through IC_50_ of 0.007 µg·mL^−1^. Further, 17 peptides fractions were identified through LC/MS/MS analysis, and seven (VW, GF, FG, SF, VY, YA, and YG) showed ACE inhibitory activity. SHR rats showed that SHB significantly decreased the systolic blood pressure in the in vivo study on SHR rats. Therefore, snail by-product hydrolysate has emerged as a new candidate for manufacturing of functional medicine and food against hypertension.

### 4.2. Anti-Inflammatory Effects

Inflammation is significantly associated with the pathogenesis of acute and chronic diseases such as multiple sclerosis, atherosclerosis, obesity, rheumatoid arthritis, neurodegenerative, and inflammatory bowel diseases [64]. Current treatment of inflammatory diseases relies largely on the use of steroidal and non-steroidal anti-inflammatory drugs (NSAIDs). NSAIDs moderate their effects by inhibiting the enzyme cyclooxygenase (COX), which catalyzes the conversion of arachidonic acid to prostaglandins (PGs) [65]. However, these NSAIDs cause an increase in blood pressure, thrombosis occurrence, the increased risk of heart attack, and also gastrointestinal erosion. Steroidal anti-inflammatory drugs also have different drawbacks, such as immunosuppressing effects.

**Figure 4:**
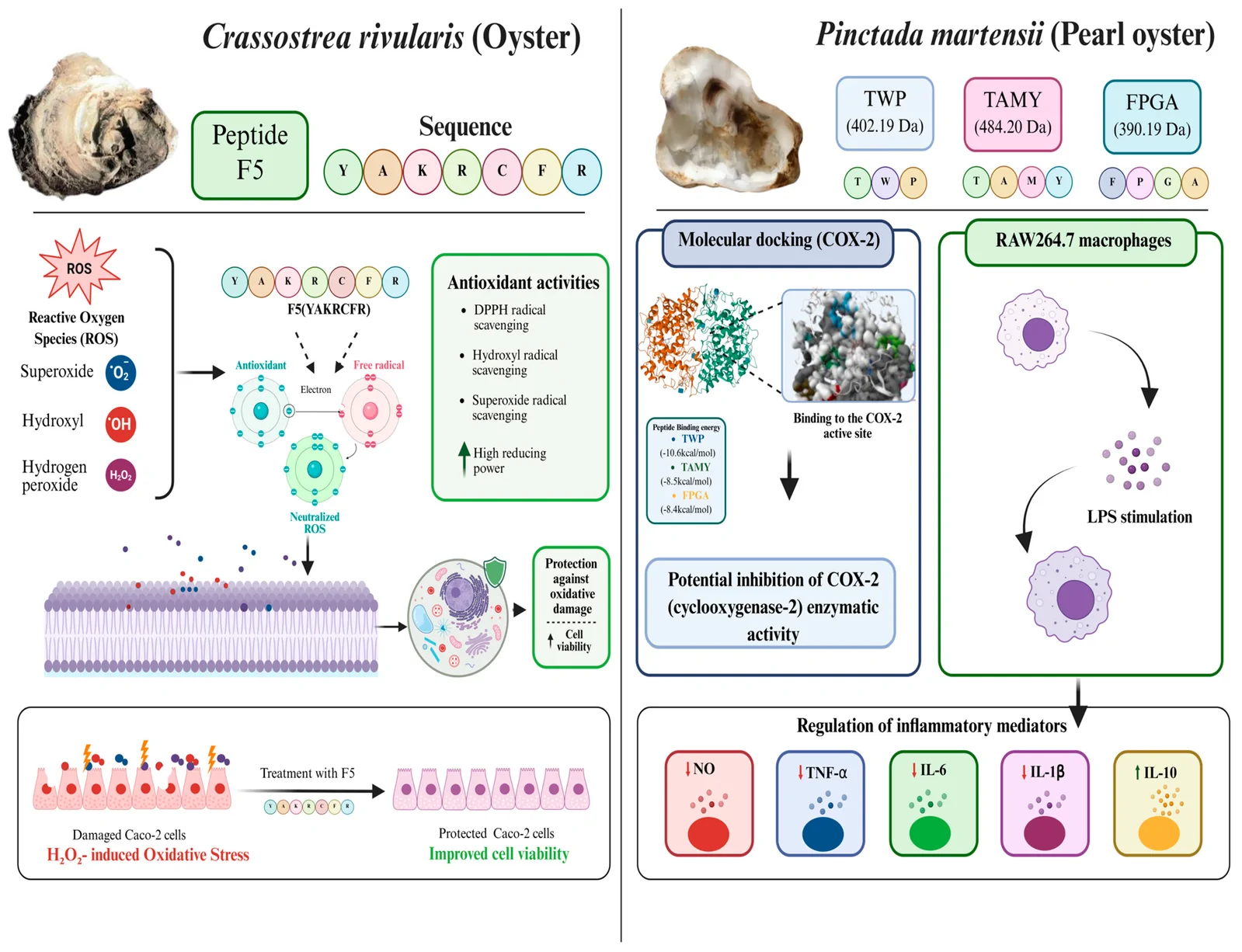
Antioxidant activity of bioactive peptides from *Crassostrea rivularis* and anti-inflammatory activity of bioactive peptides from *Pinctada martensii*.

Chen et al. [66] isolated *Arca subcrenata* muscle polypeptide, designating PGC using ion exchange, Sephadex G-50 gel chromatography and RP-HPLC. Native PAGE indicated that PGC was homogeneous and 98.9% pure. PGC showed the N terminus amino acid sequence as PSVYDAAAQLTADVKKDLRDSWKVIGGDKKGNGVA through Edman degradation. Nitric oxide (NO), produced from L-arginine by nitric oxide synthase, contributes to the cytotoxicity of activated macrophages, circulatory failure, and immune-inflammatory response. Another research by Joshi & Nazeer [54] on green mussel *Perna viridis* foot (PVF) assessed the possibility of pro-inflammatory cytokines, nitric oxide (NO) and cyclooxygenase-2 reduction in RAW 264.7 cells. The PVF was hydrolyzed with different enzymes, and anti-inflammatory activity was evaluated through HRBC Membrane Stabilization. Further, hydrolysates were separated through ultrafiltration and purified using size-exclusion chromatography. The purified peptide sequence was identified by LC-MS/MS for functional properties. Research results showed that alcalase hydrolysate exhibited anti-inflammatory activity, and a further 30–10 kDa active fraction was purified; active peak-II was identified as EGLLGDVF and showed a prominent functional role. These peptides reduced the production of pro-inflammatory cytokines, NO production and COX-2 activation and regulated COX-2 and iNOS protein expression in RAW 264.7 cells. A similar study by Shen et al. [67] isolated anti-inflammatory peptides from *Pinctada martensii* that have shown significant immunomodulatory activities in LPS-stimulated RAW264.7 macrophages. These three novel peptides, TWP, TAMY and FPGA, were shown to dose-dependently inhibit the production of pro-inflammatory mediators such as nitric oxide (NO), tumor necrosis factor (TNF- α), interleukin 6 (IL-6) and interleukin 1 (IL-1 β) and stimulate the release of the anti-inflammatory mediator interleukin 10 (IL-10). Molecular docking results also revealed that these peptides bind to important residues in the cyclooxygenase-2 (COX-2) active site, which could inhibit inflammatory pathways as described in Figure 4. Peptides found in mollusks have potential as natural anti-inflammatory agents for functional food products and drug development.

### 4.3. Antioxidant Effects

Free radicals are unstable and highly reactive and have the capability to damage cells and alter proteins, carbohydrates, lipid molecules, and DNA, which causes human diseases such as cancer and neurological, pulmonary, cardiovascular, and ocular diseases [68]. So, the use of antioxidants is crucial due to their ability to neutralize excessive free radicals or reduce free radical formation through scavenging. Natural antioxidant peptides are produced by different animal, plant, and marine species by enzymatic hydrolysis. For determination of antioxidant activity of peptides, two most common assays found are the scavenging of 2,2-diphenyl-1-picrylhydrazyl (DPPH) free radical, a stable free radical with an unpaired valence electron at one atom of nitrogen bridge and scavenging of 2,2-azinobis-(3-ethylbenzothiazoline-6-sulphonic acid) (ABTS) free radical [69]. Antioxidant peptides are produced from various marine sources such as mollusks, fish, and algae. Mollusk antioxidant peptides have a low molecular weight around 3 kDa. Pepsin is the most commonly used enzyme for the production of antioxidant peptides; these peptides can scavenge DPPH, hydroxyl, peroxyl, and ABTS radicals.

A study identified antioxidant peptides from *Meretrix meretrix* L. through simulated gastrointestinal digestion in vitro and in silico. MML hydrolysates showed protective potential against hydrogen peroxide-induced stress in Raw 264.7 cells. Under oxidative stress conditions, hydrolysates derived from the intestinal phase of digestion (MMLHs-I) significantly inhibited the reduction of cell viability, restricted the production of reactive oxygen species, and increased the activity of important antioxidant enzymes such as glutathione reductase, catalase, superoxide dismutase, and glutathione peroxidase, thereby enhancing the antioxidative defense mechanism of the cells [70]. Another study compared antioxidant peptides from two mollusks, *Tympanatonus fuscatus* var *radula* and *Pachymelania aurita peptides*, for antioxidant activity. *T. fuscatus* and *P. aurita* peptides are produced by simulated gastrointestinal digestion using trypsin, pepsin, and chymotrypsin and fractionated through a 3 kDa membrane filter. These fractions were examined for hydroxyl radical scavenging activity, ferric reducing antioxidant property, anti-lipid peroxidation, and metal chelation activity, and these showed potential antioxidant properties in all assay models [65]. Another study by Huang et al. [13] isolated five antioxidant peptide fractions (F1–F5) from oyster *Crassostrea rivularis* and evaluated antioxidant activity. Peptide F5 (YAKRCFR) showed high scavenging capabilities against DPPH, hydroxyl and superoxide radicals, and high reducing power. In addition, F5 significantly inhibited the oxidative damage of the Caco-2 cells treated with H_2_O_2_ by preserving cell viability, suggesting that F5 could be used as a natural antioxidant peptide in functional foods, nutraceuticals, etc. The peptide’s amino acid composition and capability to donate electrons or hydrogen atoms for the stabilization of reactive oxygen species (ROS) were attributed to its antioxidant activity as depicted in Figure 4.

### 4.4. Anticancer Activity

Mollusk-derived peptides are being investigated as potential anticancer leads, although most evidence remains preclinical. The peptides have anticancer activity using different interrelated mechanisms. Apoptosis is one of the primary mechanisms through which bioactive peptides exert anticancer effects. The formation of a peptide-zinc complex can be seen in oysters.

**Figure 5:**
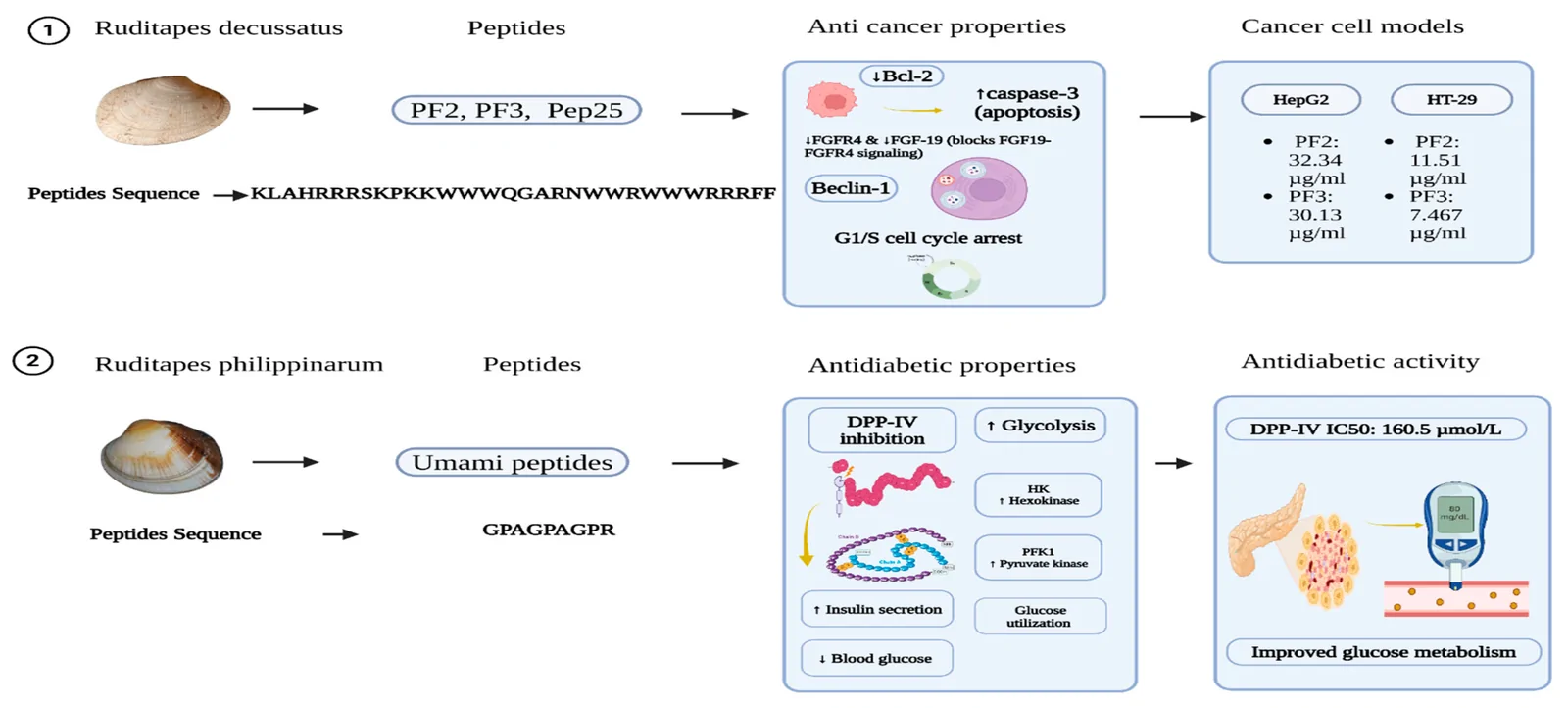
Anticancer peptides from *Ruditapes decussatus* and antidiabetic peptides from *R. philippinarum* with their molecular mechanisms and bioactivities.

Crassostrea gigas caused arrest in the S phase of the HepG2 hepatocellular carcinoma cell cycle. This prevented DNA replication and increased the levels of antioxidant enzymes, slowing down the development of single-pathway resistance. The same oyster peptide-zinc complex modulates ROS levels beyond cytotoxic thresholds in tumor cells, leading to apoptosis. Zinc also restores p53 tumor-suppressor function by increasing its bioavailability in cells by peptide chelation [71]. Hussein et al. [72] identified 135 low-molecular-weight peptides from the soft tissue of *Ruditapes decussatus* using anion-exchange FPLC. Among these, Pep25 (KLAHRRRSKPKKWWWQGARNWWRWWWRRRFF) received the highest AntiCP 2.0 prediction score (0.83); however, Pep25 was not purified and tested independently. The experimental cytotoxicity values therefore apply to the peptide fractions rather than to Pep25: PF3 produced IC_50_ values of 7.47 µg mL^−1^ in HT-29 cells and 30.13 µg mL^−1^ in HepG2 cells, while PF2 and PF3 showed comparatively low cytotoxicity toward normal human hepatocytes and VERO cells. Because no standard chemotherapeutic agent was tested under the same conditions, these values cannot establish potency relative to conventional chemotherapy. Accordingly, these fractions should be regarded as preliminary food-derived sources of anticancer lead peptides with in vitro selectivity, rather than as pharmaceutically competitive or demonstrably safer alternatives. PF2 and PF3 downregulated Bcl-2, FGFR4, and FGF19, upregulated caspase-3 and Beclin-1, and induced apoptosis, autophagy, and G1/S-phase cell-cycle arrest, as summarized in Figure 5.

Mollusk peptides inhibit the signal transduction of the immunosuppressive tumor microenvironment, involving the suppression of signaling pathways such as TNF-α, IL-6, IL-1β, and NF-κB. Oyster peptides, which have low MW, co-administered with cyclophosphamide in a Lewis lung carcinoma model, maintained the integrity of spleen and liver, increased IgG and IgM, and diminished systemically produced cytokines. There are some relevant tools, including AntiCP, Cancer PPD, and molecular docking platforms, which allow virtual screening of receptor tyrosine kinases, caspases, and cell cycle proteins, thereby speeding up the discovery of novel sequences. Despite this promise, most of the evidence is still in vitro, and clinical trials, selectivity data, and pharmacokinetic profiling are all greatly needed.

### 4.5. Mineral-Binding, Anti-Fatigue, and Metabolic Activity

Mineral-binding peptides represent a distinctive bioactivity class for mollusk-derived peptides because several edible mollusks, especially oysters, are naturally rich in trace minerals and contain acidic, basic, and histidine-containing sequences that can coordinate metal ions. In oyster peptides, zinc binding has received the most attention. More recently, a peptide derived from oyster was found to bind zinc and enhance zinc uptake and recovery of mitochondrial function by AMPK/PGC1-alpha/NRF-1/TFAM signaling, suggesting that mineral-binding peptides link the delivery of minerals to cellular energy metabolism and not only passive mineral transport [73].

Blue mussel (*Mytilus edulis*) hydrolysates have yielded two of the best-characterized calcium-binding peptides. The octapeptide LGKDQVRT, released by neutral-proteinase hydrolysis of blue mussel protein, spontaneously binds calcium at a 1:1 stoichiometric ratio via a metal-donor to metal-acceptor complex involving the Val6 residue and promotes calcium uptake across Caco-2 cell monolayers, osteoblast proliferation, and alkaline phosphatase activity [74]. A second mussel-derived nonapeptide, IEELEEELEAER (PIE), forms an analogous 1:1 calcium complex through amino nitrogen and carboxyl oxygen atoms on two glutamic acid residues, facilitates calcium uptake through Caco-2 monolayers, and simultaneously promotes osteoblast growth while suppressing osteoclast activity, indicating a coupled mineral-delivery and anti-osteoporotic function [75]. Squid extends this mineral-binding profile from bivalves into cephalopods and from calcium into zinc: Argentine squid protein peptides, produced by optimized enzymatic hydrolysis, were chelated with zinc sulfate to form stable, antioxidant-active zinc-peptide nanoparticles [76], while squid skin has independently been used as a substrate for calcium-chelating peptides evaluated in the Caco-2 absorption model [77]. At the structural level, Manila clam (*Ruditapes philippinarum*) expresses a ferritin H-like subunit (RpFeH) retaining the seven metal ligands of the ferroxidase center and the canonical iron-binding region signatures, with confirmed iron-chelating and antibacterial activity, illustrating that iron-coordinating architecture is intrinsic to calm proteins and a plausible source of iron-binding peptide fragments upon hydrolysis [78].

Anti-fatigue and metabolic activities provide a research direction for functional foods and pharmaceutical-type applications. Oyster polypeptide fractions enhanced exhaustive swimming time in mice, increased exhaustive swimming time and liver glycogen, reduced blood lactate, and modulated AMPK-related energy metabolism and were associated with changes in gut microbiota composition [79]. Together, these findings indicate that mollusk peptides may influence fatigue via their influence on energy substrate storage, lactate clearance, inflammatory tone, and microbial metabolism. Enzymes have also been targeted for metabolic regulation. Portuguese oyster (*Crassostrea angulata*) hydrolysates showed in vitro ACE and DPP-IV inhibitory activities, with pepsin hydrolysates and low-molecular-weight fractions showing stronger inhibitory effects than crude hydrolysates [80]. A subsequent computational and experimental study revealed the oyster peptide LRGFGNPPT as a candidate inhibitor of DPP-IV, which may be important to targets in type 2 diabetes (T2D) [81].

Manila clam hydrolysates provide direct evidence that DPP-IV inhibitory activity extends beyond oyster within the mollusk class. Fractionation and nano-LC–MS/MS analysis of the *Ruditapes philippinarum* hydrolysate, combined with database searching and molecular docking, identified four candidate DPP-IV-inhibitory peptides. They identified four candidate DPP-IV inhibitory peptides, FAGDDAPR, LAPSTM, FAGDDAPRA, and FLMESH, which were chemically synthesized and confirmed to inhibit DPP-IV with IC_50_ values of 168.72, 140.82, 393.30, and >500 μM, respectively, supporting their potential as functional food ingredients for diabetes prevention [82]. Zhang et al. [83] evaluated the DPP-IV inhibitory activity of 14 umami peptides from *Ruditapes philippinarum*. GPAGPAGPR (GR-9) was the only peptide with notable DPP-IV inhibitory activity (IC_50_ = 160.5 µmol/L). GR-9 retained 80.26% of its DPP-IV-inhibitory activity after simulated gastrointestinal digestion, which is higher than other peptides. In an oral glucose tolerance test in C57 mice, GR-9 reduced plasma glucose levels at 30 and 60 min after glucose injection to be significantly lower than those in the control group. Cell experiments with HepG2 showed that the GR-9 peptide exerted hypoglycemic activity by increasing the hexokinase and pyruvate kinase levels. The results indicated that GR-9 was a novel bifunctional umami peptide containing DPP-IV inhibitory and hypoglycemic activity that was resistant to digestion, indicating it should be considered as a food-derived antidiabetic peptide as depicted in Figure 5.

Abalone (*Haliotis discus*) contributes a mechanistically distinct route into metabolic regulation, acting through suppression of adipogenesis rather than enzyme inhibition. The peptide αAL14, designed from the primary sequence of a novel antimicrobial peptide purified from abalone gill, potently and dose-dependently suppressed lipid droplet accumulation during 3T3-L1 preadipocyte differentiation and downregulated the adipogenic regulators PPARγ, C/EBPα, and SREBP1. αAL14 additionally blocked insulin-stimulated PI3K/Akt signaling and its downstream mediators mTOR, GSK-3β, and Fox transcription factors, pathways that otherwise drive adipogenesis and lipid accumulation—linking an abalone-derived peptide to obesity—and diabetes-relevant metabolic regulation through a signaling axis distinct from the AMPK/PGC-1α mechanism reported for oyster polypeptides [84].

Collectively, these findings show that mineral-binding activity among mollusk peptides is broadly distributed across bivalves and cephalopods, mussel and squid. Mollusk contribute well-characterized calcium- and zinc-binding systems, and clam ferritin demonstrates intrinsic iron-coordinating capacity, while metabolic regulation spans from clam DPP-IV inhibition to an entirely distinct adipogenesis-suppressing mechanism in abalone. Direct anti-fatigue swimming-model evidence specific to non-oyster mollusks remains comparatively underexplored relative to fish-derived peptides, representing a clear gap for future investigation.

### 4.6. Gut-Health and Microbiome-Interacting Activity

Gut-health effects are particularly relevant for the development of mollusk peptide research. The orally administered peptides first interact with the gastrointestinal environment before reaching systemic targets. In cyclophosphamide-treated mice, oyster peptides enhanced intestinal immune and barrier markers, increased expression of Occludin, Claudin-1, ZO-1, Mucin-2, increased short-chain fatty acid content, and modulated gut microbiota as compared with normal mice [85]. In another cyclophosphamide-induced intestinal oxidative injury model, oyster peptides improved ileal morphology and villus structure, as well as increased antioxidant enzyme activities, decreased malondialdehyde (MDA) levels, and activated the antioxidant signaling pathway Nrf2-Keap1 in ileal tissue [86]. These results indicate that mollusk peptides play a role in the intestine by protecting epithelial integrity and redox balance. Microbiome-interacting effects also appear in inflammatory bowel disease and fatigue-related models. Oyster peptides alleviated dextran sulfate sodium-induced ulcerative colitis in mice by improving intestinal microbiota structure and inhibiting the TLR4/NF-kappaB inflammatory pathway [87].

A pentadecapeptide (RVAPEEHPVEGRYLV, designated SCSP/NJSP) purified from protein hydrolysates of the Venus clam *Cyclina sinensis* restored bodyweight, immune organ indices, and serum levels of hemolysin, TNF-α, IgA, IgG, and IgM in cyclophosphamide-induced immunosuppressed mice, while also increasing SOD and catalase activity and total antioxidant capacity and decreasing MDA in serum, indicating that the peptide protects the immune system against oxidative damage in parallel with restoring immune organ function [88]. Mechanistic follow-up work showed that this clam peptide enhances splenic lymphocyte and macrophage proliferation and phagocytosis by activating the MAPK/NF-κB and PI3K/Akt signaling pathways, elevating phosphorylated p38, ERK, JNK, PI3K, and Akt, upregulating IKKα, IKKβ, and NF-κB p50/p65. Downregulated IκBα demonstrated a signaling route distinct from, but convergent with, the barrier- and microbiota-focused mechanisms reported for oyster peptides [89]. Thick-shelled mussel (*Mytilus coruscus*) protein hydrolysate has independently yielded 60 low-molecular-weight (428.30–935.46 Da) immunomodulatory peptides identified by LC-MS/MS, among which LH-6 (LVVLGH) significantly activated RAW264.7 macrophages, promoting proliferation, phagocytosis, and secretion of NO, IL-6, IL-1β, and TNF-α, extending the immune-priming activity documented for clam and oyster peptides to a mytilid bivalve [90].

In the ulcerative colitis model, abalone contributes a close parallel to the DSS-induced colitis findings reported for oysters. Collagen peptide extracted from abalone viscera (AVCP), administered orally at 300–900 mg kg^−1^ day^−1^ to DSS-induced colitis mice, alleviated weight loss, colon shortening, and disease activity index escalation; reduced pathological colon damage, splenic edema, and thymic atrophy; lowered serum IL-1β, IL-6, TNF-α, IL-17A, and myeloperoxidase; and improved antioxidant capacity (increased SOD and GSH-Px, decreased MDA). 16S rRNA sequencing further showed that AVCP reshaped the gut microbiota, altering the abundance of taxa associated with active ulcerative colitis (e.g., *Escherichia-Shigella*, *Bacteroides*, *Parasutterella*, *Muribaculaceae*, *Rikenellaceae*) [91]. These findings support oral activity of the peptide preparation in mice but do not establish that individual sequences remained intact, crossed the intestinal epithelium, or directly mediated the effects. Local mucosal or microbiota interactions and bioactive digestion products may also contribute. Future studies should integrate simulated gastrointestinal digestion, epithelial transport and sequence-tracking assays, pharmacokinetic analyses, and human trials.

## 5. Structure–Function Relationships

The function of mollusk-derived peptides is not determined by a single descriptor but by the combined effects of primary sequence, chain length, net charge, hydrophobicity, secondary structure and post-translational modifications (PTMs). For food-derived mollusk peptides, such as oyster, mussel, clam, scallop and squid protein hydrolysate peptides, activity is often governed by short linear sequences enriched in hydrophobic, aromatic or basic residues. For venom-derived mollusk peptides, especially cone snail conopeptides, the same structure–function logic is extended by extensive PTMs, including disulfide bonding, C-terminal amidation, gamma-carboxyglutamate (Gla), hydroxyproline, bromotryptophan and D-amino acids, which generate highly constrained receptor-binding scaffolds. Therefore, a structure–function framework for mollusk peptides should integrate both food peptide chemistry and the more pharmacologically specialized conopeptide paradigm [92].

### 5.1. Primary Structure and Bioactivity

The primary sequence is a major determinant of peptide activity because it governs side-chain chemistry, molecular size, charge distribution, hydrophobicity, and conformational potential. In antioxidant peptides, residues capable of hydrogen or electron donation, radical stabilization, proton transfer, or metal chelation may contribute to activity. The imidazole side chain of His can participate in proton transfer and transition-metal binding, while the aromatic side chains of Tyr and Trp can stabilize radical intermediates. Pro may indirectly influence activity by modifying peptide conformation, solvent exposure, and resistance to proteolysis [93]. However, these residues are neither equally important nor independently predictive of antioxidant potency. Activity depends on residue identity and position, sequence context, peptide length, hydrophobicity, and assay conditions rather than composition alone (Supplementary Table S2). This positional dependence is illustrated by ω-conotoxin GVIA: replacing Lys2 with Ala increased the IC_50_ for N-type calcium-channel binding from 0.15 to 5.5 nM, whereas replacing Lys24 caused no significant change [94]. This example demonstrates that the same residue can contribute differently depending on its local conformation and target interactions.

For ACE-inhibitory peptides, hydrophobic and aromatic residues are frequently enriched at the C-terminal region, where they can fit into the hydrophobic pockets of the ACE catalytic site. Pro is especially important because it increases resistance to digestive peptidases and frequently appears in potent ACE-inhibitory sequences. Aromatic residues such as Tyr, Phe, and Trp, as well as branched-chain residues such as Leu, Ile, and Val, often strengthen enzyme–peptide interactions. Basic residues, including Arg and Lys, may contribute electrostatic interactions with negatively charged residues around the active site. This explains why many active ACE-inhibitory peptides from marine protein hydrolysates are short, hydrophobic, or amphiphilic sequences rather than highly polar peptides. In mollusk systems, ACE-inhibitory peptides have been reported from oyster sauce and squid gelatin hydrolysates, supporting the relevance of this food-peptide SAR model to mollusk-derived materials [95].

Net charge and charge distribution are especially important for antimicrobial activity. Most membrane-active antimicrobial peptides are cationic and amphipathic. Positive charges, especially Arg and Lys, promote electrostatic binding to negative charges on the bacterial envelope, such as lipopolysaccharides, lipoteichoic acids, and acidic phospholipids. The guanidinium group of Arg is able to form bidentate hydrogen bonding and cation-pi interactions, which often provide a stronger association with the membrane than the ammonium group of Lys. But there needs to be a balance of charge; increasing positive charge can strengthen peptide binding to bacterial membranes, but excessive charge may reduce selectivity and increase cytotoxicity. Thus, the spatial distribution of the cationic residues on one side of a helix or beta-sheet is often more predictive than the charge alone [96].

The contribution of hydrophobicity is also activity-dependent. For antimicrobial peptides, hydrophobic residues enable insertion into lipid bilayers after electrostatic interaction with the bacterial surface. In the case of ACE-inhibitory peptides, hydrophobic residues improve accommodation in the ACE hydrophobic pockets. In antioxidant peptides, moderate hydrophobicity can facilitate the accessibility of the lipid radicals and oxidizing interfaces, and aromatic hydrophobic residues can stabilize radical products. However, high hydrophobicity may decrease aqueous solubility, increase aggregation, and increase hemolytic or cytotoxic activity. Hence, an optimal hydrophobicity–amphipathicity balance is more useful than maximization of H [97].

### 5.2. Secondary Structure and Bioactivity

Secondary structure converts primary sequence information into three-dimensional functional surfaces. The conformations of alpha-helical, beta-sheet and random-coil structures can support activity through different mechanisms. In antimicrobial peptides, amphipathic alpha-helices are among the best characterized motifs. These peptides tend to be fairly unstructured in water but form helices when they encounter microbial membranes. Helix formation allows segregation of hydrophobic residues onto one side of the helix, and of cationic/polar residues onto the opposite side of the helix, forming an amphipathic surface that initially binds to the negatively charged membrane and subsequently perturbs lipid packing within the membrane. This mechanism is what accounts for the fact that in many cases, antimicrobial activity is related to hydrophobicity, charge distribution and helicity [98].

Particularly, beta-sheet structures are prevalent in antimicrobial peptides and conopeptides, which are cysteine-rich. Mussels make myticins, 40-residue cysteine-rich peptides with four intramolecular disulfide bonds, which are compact and antimicrobial, and represent an example of how mollusk AMPs can attain stability and antimicrobial activity through disulfide-constrained folds, not just a simple linear helix. Cysteine-stabilized structures also play a role in maintaining an active surface in the hemocytes and plasma of mussel through the activity of those mussel defensins and related peptides. Such scaffolds are advantageous in marine environments because they resist proteolysis, salinity changes, and microbial enzymes [99].

Random coil/flexible regions are not considered inactive. Many short food-derived antioxidant and ACE-inhibitory peptides need not adopt stable secondary structures in solution; instead, their activity relies on the exposure of functional side chains and conformational adaptability at enzyme, radical or metal-binding interfaces [95]. Similarly, localized flexibility in some conopeptides is employed to maximize receptor recognition, and disulfide bonds provide stability in the global scaffold. So, the secondary structure–activity correlation is best thought of as context-dependent; while membrane-disrupting peptides can gain from amphipathicity that is induced by the membrane, enzyme-inhibiting or receptor-binding peptides may prefer either a flexible fit to the receptor or a ‘lock-and-key’ presentation of the pharmacophoric residues [100].

### 5.3. Post-Translational Modifications and Their Effects

PTMs are especially important in cone-snail venom peptides, which are among the most extensively modified molluscan peptides, as described in Table 3. Such changes increase the chemical diversity beyond the 20 standard amino acids and may influence folding, stability, receptor selectivity, and pharmacological potency. PTMs have not been studied systematically in food-derived mollusk peptides but may affect the bioactivity and stability of the peptides during processing, that is, terminal modifications, oxidation, deamidation, and phosphorylation. Therefore, PTMs should be integrated into a complete structure–function model, rather than being secondary annotations [101,102].

The most common PTM-like covalent constraint found in cysteine-rich mollusk peptides are disulfide bonds. In conotoxins, disulfide bonds typically create tight 3D scaffolds, with two to four bonds. These bridges are important for preserving the spatial disposition of receptor binding residues, for the resistance to proteolysis, and for the conformational entropy constraint in target binding. Disulfide connectivity is no longer just a stability factor; it can determine pharmacological activity and receptor selectivity. Distinct functions can be encoded from the same primary sequence (disulfide isomers); for example, globular and ribbon disulfide isomers of Conus geographus alpha-conotoxins show markedly different potency and subtype selectivity at human nicotinic acetylcholine receptors, with globular isomers generally more potent but ribbon isomers retaining or, in some cases, matching activity depending on the conotoxin’s loop-length subgroup [103]. Multiple disulfide bonds also stabilize antimicrobial folds in the extracellular marine environment, such as in bivalve AMPs, myticins, and defensins. Mollusks are a rich source of antimicrobial peptides (AMPs) that function as key effectors of innate immune defense against bacterial, fungal, and viral pathogens. The Mediterranean mussel (*Mytilus galloprovincialis*) produces 40-residue, cysteine-rich myticins containing four intramolecular disulfide bonds that are always present in hemocytes and plasma. In addition to antibacterial, chemotactic, and wound-healing activities, the myticin C isoform inhibits the replication of ostreid herpesvirus 1 (OsHV-1) in oyster hemocytes, and its sequence exhibits significant allelic diversity indicative of positive selection, suggesting a role in host–pathogen coevolution [20]. Another example is hdMolluscidin, a distinct 46-residue, 4.7 kDa antimicrobial peptide purified from the gill of abalone *Haliotis discus*. This peptide showed broad-spectrum, potent activity against both Gram-positive and Gram-negative bacteria with minimal hemolytic activity, and its primary sequence later served as the structural template for the anti-adipogenic peptide αAL14 [104].

One of the most unique modifications to conopeptides is gamma-carboxyglutamate (Gla). It is generated by vitamin K-dependent gamma-glutamyl carboxylation and gives peptides the ability to bind divalent cations. In the Gla-containing NMDA-receptor antagonists conantokin-G and conantokin-T, metal binding influences α-helical structure and biological activity. Experimental studies have demonstrated that individual Gla residues contribute differently: In conantokin-G, the substitution of Gla4 by Ala led to a complete loss of antagonist activity in an MK-801 binding assay, and the substitution of Gla3 reduced the potency by about 20-fold, whereas Gla10 and Gla14 were more important for cation-dependent helical stabilization than for direct receptor inhibition [105]. The PTM in this example can serve as a structure switch as well as a functional pharmacophore.

Hydroxyproline (Hyp), a product of proline hydroxylation, affects local geometry, hydrogen-bonding capabilities, and hydration of peptides. Hyp can help stabilize turns or polyproline-like conformations and influence folding efficiency and receptor recognition in conopeptides. Hydroxylation may affect both activity and stability of pro-containing ACE-inhibitory peptides due to the change of polarity and conformational preference of hydroxylated Pro, which is common in food-derived ACE-inhibitory peptides. Hence, the presence of Hyp in marine peptides must be confirmed by experimental means and not just predicted based on the occurrence of Pro [101,102].

Residue epimerization results in D-amino acids that have a significant impact on peptide stability and recognition. D-residues are not easily degraded by many proteases, which recognize the L-amino acid substrate, and may provide new turn conformations or orientations at the receptor. D-amino acids have been found in the conopeptides, which belong to contryphan and related families and are responsible for conformational uniqueness and bioactivity. Selective D-substitution as a design strategy is very effective to increase stability; however, if a key pharmacophore residue is inverted, it will no longer bind to the target. Conopeptides often undergo C-terminal amidation, and, moreover, this could be relevant to synthetic optimization of food-derived peptides as well. Amidation of a C-terminal carboxylate neutralizes the negative charge, alters net charge and dipole moment, and can enhance receptor/ion-channel affinity and provide protection from carboxypeptidases. In conotoxins like ziconotide and many of the alpha-conotoxins, amidation is a part of the mature bioactive form. Amidation could improve stability and binding properties of linear food peptides but also affect solubility and regulatory status and should thus be studied in conjunction with native acid forms. Peptides can be protected against degradation by aminopeptidases by N-terminal cyclization to pyroglutamate (pGlu), which also lowers the N-terminal positive charge. In addition to this, this modification could have an impact on receptor binding by restricting the N-terminal region and increasing the circulating stability. In a wider sense, cyclization (head-to-tail cyclization and side-chain stapling) can also be a rational design strategy given the decrease in conformational entropy and the concomitant enhancement in resistance to proteolysis [102].

Bromotryptophan (BrTrp) is a marine-associated-halogenated residue that has been detected in cone snail venom peptides. Original biochemical and mass spectrometric studies revealed the presence of peptides containing 6-bromotryptophan in Conus imperialis and Conus radiatus, and further studies have shown the presence of conotoxin sequences containing bromotryptophan in *Conus amadis* venom [106]. The bromination leads to an enlargement of the indole side chain and an increase in its hydrophobicity and polarizability, which could change hydrophobic contacts, pi-pi stacking, and/or cation–pi interactions. For many of the conopeptides containing BrTrp, there is too little functional data available to consider this modification as an activity enhancement proof of concept; rather, it should be viewed as a potential modulator for receptor interaction and peptide diversity. Another review by Mardirossian et al. [107] described halogenated amino acids in antimicrobial peptides (AMPs), with a focus on bromotryptophan (BrTrp). Five BrTrp isomers were identified in marine sponges and invertebrates, formed by post-translational bromination via bromoperoxidases. Although free BrTrp showed little bioactivity, BrTrp-containing peptides, including jaspamide, styelin D, and various conotoxins, displayed strong antimicrobial and pharmacological effects. In most ribosomally synthesized AMPs (e.g., HFIAPs, hedistin, centrocins), bromination occurred at position 6 of the indole ring and improved protease resistance but was not essential for antimicrobial activity. An exception was the lantibiotic NAI-107, where halogenation directly boosted potency against multidrug-resistant Gram-positive bacteria. The review also showed that synthetic halogenation of peptides and peptidomimetics can enhance antimicrobial activity, sometimes without increasing toxicity, supporting its use in AMP drug design.

Further chemical modifications are provided by O-glycosylation and tyrosine sulfation. O-glycosylation has been shown to enhance solubility, to confer resistance to proteolysis, and to affect tissue distribution, and sulfation adds a powerful negative charge that can alter electrostatic interactions with receptors or ion channels. Relatively little is known about these PTMs compared to disulfide bonds, amidation or Gla in conopeptides, but they are important to include because mollusk peptides are often products of secretory pathways where PTM enzymes can modify and diversify the final products [101].

Peptide bioactivity is inseparable from molecular structure, yet current understanding of structure–function relationships in mollusk-derived peptides remains largely fundamental and descriptive. The examples discussed above (Supplementary Table 3 and Table S2) show that similar structural changes can have effects ranging from negligible to a complete loss of activity, depending on the molecular scaffold. This context dependence arises because individual residues function within a specific conformational landscape, solvation environment, and target-binding geometry. Consequently, a substitution tolerated in one scaffold may destabilize a critical turn or abolish receptor engagement in another. Cross-study comparisons are further limited by differences in assay design and reporting standards, while peptide activity itself emerges from the integrated molecular context rather than from independently additive residue contributions. Developing a predictive framework will therefore require standardized, multidimensional datasets that combine experimentally resolved structures, quantitative potency values such as EC_50_, and pharmacokinetically relevant properties, including protease resistance and bioavailability. Such curated datasets could support machine learning approaches to identify functionally important residues and prioritize candidate peptides for experimental validation.

## 6. Applications

### 6.1. Food Industrial Applications of Mollusk-Derived Peptides

The potential use of mollusk-derived peptides in the food industry is influenced by their ability to improve texture and mechanical properties and their antibacterial and antioxidant activities. Gelatin and collagen, both marine-derived proteins, have long been used as food ingredients because they form gels and films and improve texture and water-holding capacity. The gel-forming and water-holding capacity of marine collagen in meat products has been attributed to enhanced bond formation and amino acid interactions with other proteins. For example, the incorporation of cod skin collagen into surimi gel has been shown to promote ionic, disulfide, and hydrogen bond formation, resulting in improved gel strength and water-holding capacity, likely due to enhanced interactions between charged amino acids and surimi myofibrillar proteins [108]. Similarly, Sousa et al. [109] demonstrated the substitution of pork backfat with hydrolyzed collagen in Frankfurt-type sausages. A 50% collagen replacement improved aroma, protein profile, oxidative stability, textural qualities, and water-holding capacity relative to sausages composed of whole pork meat. Mollusk-derived collagen is receiving heightened interest for its possible role as an emulsifier in diverse food products, such as ice cream, chocolate, yogurt, and butter. Collagen hydrolysates have demonstrated the ability to maintain the emulsion characteristics of butter and chocolate sauce, thereby greatly prolonging their shelf life [110].

Mollusk-derived collagen has a wide range of applications in beverages because it can enhance nutritional and functional properties, is highly soluble, and has low viscosity. Its antioxidant activity may also contribute to shelf life extension. The incorporation of ossein derived from salmon scales into chrysanthemum tea as a research study showed that the sensory, antioxidant and physicochemical properties of the tea changed during 6 months of storage. The findings showed that the addition of collagen increased the α-amino group, pH, turbidity and browning index as well as improved the beverage’s antioxidant properties and extended its shelf life for six months at 30 °C [111]. Mollusk collagen is used widely in the packaging of meat, poultry, and marine products and is characterized by its native triple-helical structure and fibril networks. Its use as an artificial casing is particularly valued for its renewable, biodegradable and biocompatible properties. The packaging materials based on collagen provide excellent moisture and oxygen barrier properties as well as mechanical, sensory and organoleptic properties.

Bhuimbar et al. [112] developed collagen-chitosan antibacterial food packaging films incorporating pomegranate-peel extract. The films inhibited the tested foodborne pathogens (*Bacillus saprophyticus* LNB 333F5, *Bacillus subtilis* NCIM 2635, *Salmonella typhi* NCIM 2501 and *Escherichia coli* NCIM 2832) and showed reduced water-vapor permeability. Song et al. [113] studied the use of mollusk skin collagen as an active packaging material. The findings indicated that collagen/zein electrospun films containing gallic acid significantly inhibited the degradation of tilapia muscle, hence extending its shelf life. An edible film was formulated with 0.5% lysozyme and 4% collagen (*w*/*v*), demonstrating optimal efficacy in inhibiting bacterial growth while maintaining the freshness of salmon filets (*Salmo salar*). Ben Slimane & Sadok [114] demonstrated that a biofilm composed of *Mustelus mustelus* collagen and chitosan exhibited enhanced UV barrier characteristics and antioxidant activity relative to chitosan alone. This biofilm shows potential as a sustained bioactive substance for the preservation of nutraceutical items.

Beyond conventional collagen applications, peptide-rich hydrolysates from oysters, clams, mussels, and other mollusks have been developed as functional-food and dietary-supplement ingredients, particularly in Japan and China, with related products also marketed in Europe [4]. Available formats include powders, tablets or capsules, beverages, jellies, and fortified foods. Their commercialization demonstrates the feasibility of producing food-grade mollusk peptide ingredients while supporting nutrient delivery, bioactivity, digestibility, and processing-by-product valorization. However, most commercial products remain complex hydrolysates or peptide-rich extracts standardized by total peptide content and molecular-weight distribution rather than by individual active sequences. Sequence-defined peptides may offer more consistent identity and dosing, facilitate mechanism-based formulation, and enable optimization for specific functions.

Collagen and gelatin are mainly valued for their structural and techno-functional properties in food matrix. These properties include gelation, emulsification, film formation, and water-holding capacity. They mainly result from the structural characteristics of collagen and gelatin. Therefore, most collagen sources show similar functional properties, regardless of their exact amino acid sequences. However, mollusk peptide hydrolysates are increasingly studied for their sequence-specific physiological activities rather than their structural functions. In silico peptidomic screening of oyster proteins has identified individual peptide sequences with predicted antidiabetic, antihypertensive, anti-inflammatory, antimicrobial, and anticancer activities. These activities are associated with the peptides’ primary structures rather than simply with the presence of collagen or gelatin [115]. Structure–activity studies of oyster-derived peptides have further shown that bioactivity depends on specific molecular characteristics. These characteristics include low molecular weight, the presence of particular hydrophobic or charged amino acid residues, and specific secondary-structure motifs. Such characteristics can determine the potency of a peptide against a particular biological target. They are different from the structural properties responsible for gel or film formation [38]. A study on hard-shelled mussel-derived high Fischer ratio oligopeptides showed significant hepatoprotective effects against acetaminophen-induced liver injury in mice. They reduced serum ALT and AST levels and improved liver index and histopathological damage. The peptides also increased glutathione levels and the activities of SOD and CAT. These effects were associated with activation of the Nrf2 pathway and suppression of inflammatory cytokines, including TNF-α, IL-1β, and IL-6 [116]. Peptides derived from pearl matrix proteins have demonstrated ACE-inhibitory activity. Another study on abalone-derived peptides used virtual enzymatic hydrolysis and molecular docking to identify potential antioxidant sequences. Ten peptides showed strong inhibition of the Keap1-Nrf2 interaction, with DEDEDEDK showing the highest antioxidant activity. In an AAPH-induced oxidative damage model, DEDEDEDK reduced reactive oxygen species and increased SOD and CAT activities and glutathione levels. These results indicate its potential to protect cells against oxidative damage [12]. These findings indicate that the bioactivity of mollusk proteins can be linked to specific and identifiable peptide sequences. This differs from conventional collagen and gelatin hydrolysates, which are generally characterized by total peptide content and molecular-weight distribution rather than by individual active sequences. Therefore, mollusk-derived peptides may offer greater potential for mechanism-based and precision-nutrition applications. Their role is distinct from the mainly structural and textural functions that collagen and gelatin currently provide in food systems.

### 6.2. Pharmaceutical Development of Mollusk-Derived Peptides

Mollusks have been recorded in Chinese, Mediterranean, Latin American and African medical systems as remedies for ocular disorders, wounds, inflammation, gastrointestinal symptoms and nervous-system complaints [117]. Several traditional or ethnomedical practices still provide plausible entry points for peptide/protein-oriented drug discovery. Pearl nacre and mother-of-pearl preparations, traditionally used for calming, ocular and tissue-repair indications, contain an organic matrix of shell proteins and peptides in addition to aragonite; modern reviews of nacre and pearl powder show that matrix proteins can be extracted, purified and tested for bioactivity, while water-soluble nacre factors have been reported to promote cutaneous wound repair by improving fibroblast and angiogenic functions [118,119]. These findings do not prove that traditional sedative or ophthalmic effects were peptide-mediated, but they do support the view that shell organic matrices are more than inert minerals and may provide proteinaceous biomaterial leads.

A second relevant example is gastropod mucus. The secretion of slugs and snails has been employed in external wound treatment in several folk-medicine traditions, and the biological activity is not related to any one unique small molecule but to mucins, glycoproteins, antimicrobial factors and peptide-like components. In the same vein, a new anti-bacterial wound-healing peptide called Healitide-GP1 was recently tested in vitro for combined antimicrobial and repair-promoting activity, indicating that the discovery of mucus-inspired or mucus-derived peptides could evolve into a feasible approach to wound-care drug design [120].

Molluscan venoms and proteinaceous defense systems represent the best peptide- specific precedents for clinical translation. The synthetic version of omega-conotoxin MVIIA, found in the venom of Conus magus, paved the way for the development of a mollusk venom peptide into an approved medicine by selectively blocking neuronal N-type calcium channels for severe chronic pain [121]. Although ziconotide represents a major clinical success, it also illustrates important limitations of peptide therapeutics. Its specialized administration route and the risk of neurological and psychiatric adverse effects require slow, individualized dose titration and close clinical monitoring. Sea hares and related heterobranch gastropods provide another translational route because depsipeptides and peptide-derived cytotoxins, including dolastatin and kahalalide scaffolds, have influenced anticancer drug development [122,123]. These examples support retaining sea-hare-derived peptide and depsipeptide cases in the pharmaceutical section while excluding non-peptidic secondary metabolites from the peptide evidence base.

Proteinaceous defense and adhesion systems offer a last link between traditional wound-care functions and modern development. Mussel adhesive proteins are DOPA-rich proteins evolved for wet adhesion; recombinant mussel adhesive proteins have been developed as cell-adhesion biomaterials and bulk adhesives, demonstrating a protein-based translational route relevant to wound closure, tissue fixation and regenerative materials [124,125]. In parallel, bivalve innate immune peptides such as myticin, mytilin and mussel defensins provide antimicrobial and antiviral leads: myticin was isolated from *Mytilus galloprovincialis* haemocytes and plasma as a cysteine-rich antimicrobial peptide, and myticin C later showed antiviral and immunoregulatory activity against mollusk and human herpesviruses [20].

Regulatory translation of mollusk-derived peptides depends not only on their biological activity but also on their intended use, route of administration, and claim wording. Peptides marketed with general physiological-support claims may follow food, dietary-supplement, health-food, or novel-food pathways, whereas claims to prevent or treat hypertension, diabetes, inflammatory diseases, or cancer generally trigger drug or medicinal-product regulation. The likely regulatory classifications and principal evidence requirements for each bioactivity in the United States, European Union, and China are summarized in Supplementary Table S3.

### 6.3. Cosmetics and Cosmeceutical Applications of Mollusk-Derived Peptides

Beyond food and pharmaceutical applications, marine-derived peptides are also emerging as active ingredients in the cosmetics and cosmeceutical sector. A recent study by Yu et al. [126] investigated the anti-aging effects of jellyfish collagen peptides on skin using cytotoxicity assessment, in vitro cell experiments (HFF-1 fibroblasts and HaCaT keratinocytes), a zebrafish tail-fin wrinkling model, and mechanistic assays targeting ROS, inflammation, and apoptosis. The peptides showed no cytotoxicity up to 250 μg mL^−1^ and reduced H_2_O_2_-induced intracellular ROS to 85% of the H_2_O_2_-only level. In the zebrafish UV-wrinkling model, the peptides inhibited tail-fin shrinkage in a dose-dependent manner, with inhibition rates of 31.5%, 62.6%, and 96.8% at 10, 100, and 250 μg mL^−1^, respectively, and at 10 μg mL^−1^ significantly increased type I and type III collagen mRNA by 51.4% and 24.8%. In LPS-stimulated HaCaT cells, the peptides suppressed the pro-inflammatory cytokines IL-6 and TNF-α by up to 23.1% and 16.2% (at 250 μg mL^−1^), and Western blot analysis showed reduced phosphorylated NF-κB/p65, caspase 9, caspase 3, and Bax protein levels, indicating suppression of NF-κB-mediated inflammatory and apoptotic signaling alongside a corresponding reduction in early-apoptotic cells. Similarly, salmon skin gelatin and its hydrolysates reduced UV-induced skin damage by increasing antioxidant enzyme levels while also enhancing immune function and promoting collagen content, as reflected by elevated hydroxyproline levels in treated skin [127]. A review by Badilli et al. [128] highlighted the growing incorporation of peptides, biotic ingredients, and marine biopolymers into anti-aging cosmetic products. These peptides are widely valued for enhancing fibroblast proliferation and collagen synthesis, supporting skin barrier function, and reducing hyperpigmentation [128]. These peptides reduce oxidative stress, reduce inflammation, and support collagen production. As demand for naturally derived skincare ingredients grows, mollusk-derived peptides warrant further evaluation for cosmetic applications.

## 7. Challenges and Future Perspectives

**Figure 6:**
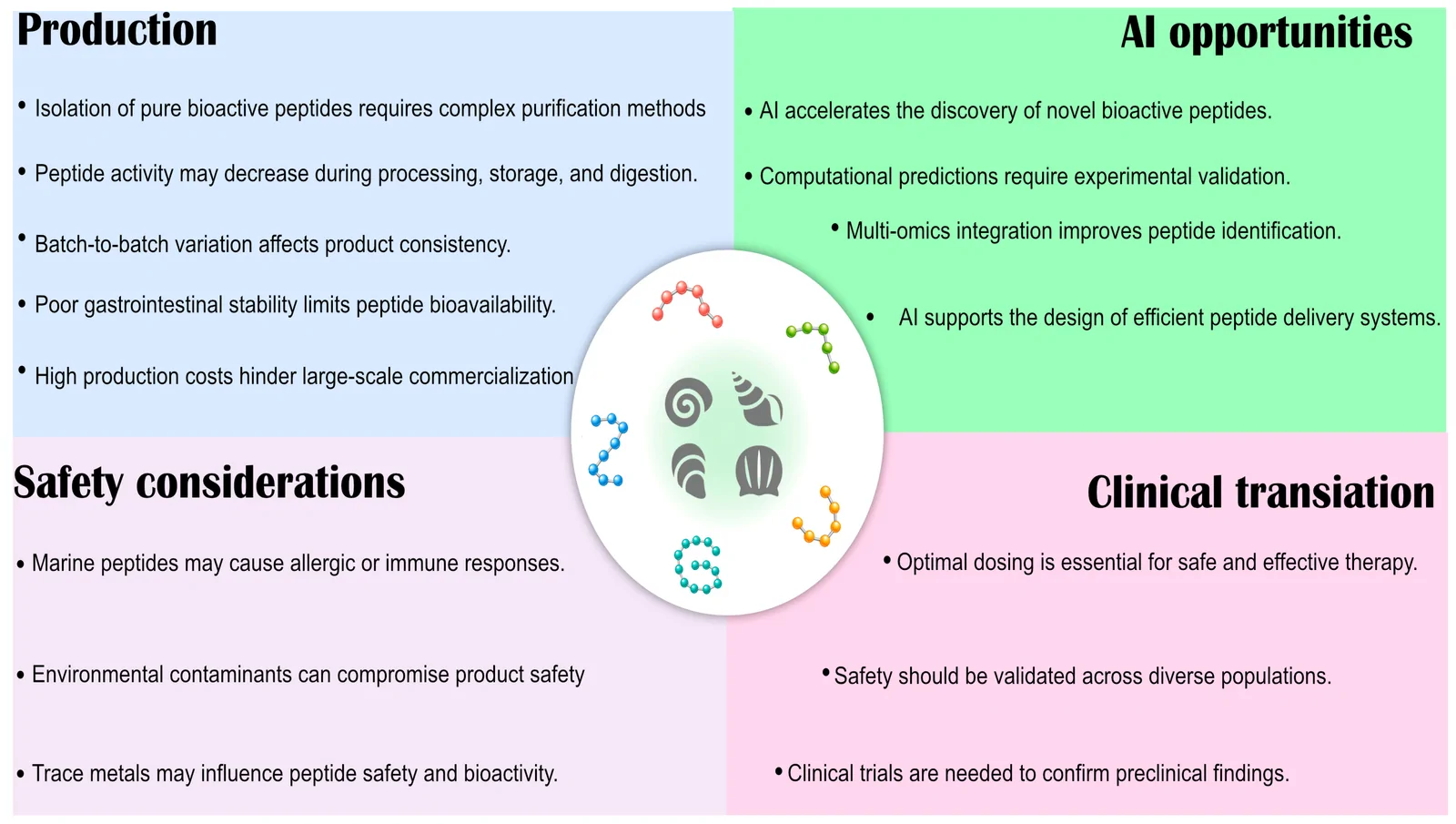
Challenges and opportunities in mollusk-derived bioactive peptide development.

Despite the immense potential of mollusk-derived bioactive peptides, several challenges hinder their large-scale utilization and commercial applications. The industrialization of mollusk-derived peptides is constrained by the interdependence among scalable production, defined product identity, reproducible efficacy, and demonstrable safety and regulatory compliance. Accordingly, the following sections systematically examine the key challenges and feasible breakthroughs across four dimensions, including production and economic feasibility, dosing and safety, clinical translation, and AI-enabled innovation (Figure 6).

Traditional extraction procedures, such as solvent-based techniques including acetic acid treatment, are unfeasible for marine sponge collagens because of their insolubility. Alkaline solubilization influences amino acid composition, the secondary structure of proteins, and the solubility of mollusk-derived protein isolates (FPIs), hence impacting their functional and therapeutic efficacy. Several studies have investigated enzymatic extraction and reported potential improvements in collagen yield. The elevated expense of enzymes impedes the industrial-scale use of enzymatic procedures. Despite the cost-effectiveness of microbial fermentation, its industrial application for mollusk-derived bioactive peptide production is hindered by low peptide yield and insufficient specificity in peptide creation [51,129].

Glycoside side chains and protein–polysaccharide interactions make it challenging to extract protein from mollusks, and the broader challenges of scalability, standardization, and cost for mollusk-derived proteins and peptides remain a significant barrier to commercialization across the wider marine protein sector [130]. Enhancement of production by alkaline solutions with reducing agents like β-mercaptoethanol is limited and precludes food applications. Food-grade reducing agents such as NAC (*N*-acetyl-L-cysteine) may improve compatibility with food applications, subject to formulation-specific safety and regulatory assessment.

The purification of target mollusk-derived peptides for laboratory-scale assessments generally uses conventional methods like membrane separation, dialysis, gel filtering, ion-exchange chromatography, and reverse-phase high-performance liquid chromatography (RP-HPLC) [129]. However, it is difficult to extract active peptides derived from mollusks from hydrolysates and fermented extracts because of the small amounts produced. Advanced separation methods, including ultrafiltration and chromatography, remain expensive and require further optimization for large-scale production. Before being used as functional food and medicinal ingredients, the toxicity and allergenicity of marine peptides should be examined. Stability and bioavailability are critical issues for peptides. Peptides can be degraded during processing, storage and digestion, leading to a decrease in their functional activity. Thus, there is an urgent need to develop delivery systems, including nanoemulsions, nanoliposomes, and polymeric nanoparticles, to improve peptide stability and bioavailability. Nevertheless, some existing extraction and purification processes may remain suitable for low-volume, high-value niche products, where higher unit costs can be tolerated; however, they are not yet economically suitable for large-volume, cost-sensitive applications [131].

Safety assessment, particularly evaluation of peptide interactions and behavior in food matrices and the human body, is a key factor for their efficient application in food and therapy. Although many mollusk peptides have been screened for bioactivity, their molecular targets, mechanisms, and metabolic pathways remain incompletely understood. Therefore, bioinformatics and computational approaches, such as molecular docking and in silico screening, are now being used to identify peptide sequences; in particular, such models using artificial intelligence can improve the identification of novel bioactive peptides and predict their activity [132]. These AI-driven pipelines generally follow a structured workflow. First, peptide sequence data are curated from bioactivity databases and converted into physicochemical feature representations, such as hydrophobicity, net charge, and amphipathicity. These features are then used to train predictive models, ranging from classical machine learning classifiers to deep learning architectures, including convolutional neural networks, which detect local sequence motifs, and graph neural networks, which capture more explicit structural relationships [132]. Once trained, these models can rapidly screen large peptide sequence spaces to predict candidates with specific bioactivities, including antimicrobial, antioxidant, anti-inflammatory, and anticancer activity, substantially narrowing the pool requiring experimental validation. Beyond activity prediction, generative AI models are increasingly being used to design novel peptide sequences optimized for a target bioactivity profile. Despite these advances, AI-based predictions still require experimental validation, as data heterogeneity, model overfitting, and the difficulty of extrapolating findings across species remain persistent limitations [133]. For mollusk-derived peptides specifically, integrating AI-based sequence-activity modeling with molecular docking could similarly accelerate the identification of novel bioactive candidates, representing a promising direction for future research. Future research should prioritize validation in human clinical trials, regulatory evaluation, and the development of novel functional food ingredients and/or new pharmaceuticals for treating a wide range of life-threatening disorders.

## 8. Conclusions

Mollusk-derived peptides are a source of valuable bioactive peptides that have not yet been fully explored for their use in functional foods, nutraceuticals, food preservation, cosmeceuticals and health-related products. Cosmeceuticals represent a promising direction for future research on mollusk-derived peptides. Protein-rich mollusk tissues, such as the mantle, adductor muscle and foot, as mentioned in this review, are useful as substrates for production of bioactive peptides. Enzymatic hydrolysis is the most widely used method for releasing peptides with desired structures and functions. Furthermore, the use of fractionation, purification and structural characterization techniques such as ultrafiltration, chromatography, mass spectrometry and bioinformatics tools have aided in the identification of peptides with antioxidant, antihypertensive, anti-inflammatory, and other biological activities. However, the progress of the application of mollusk-based peptides is still in its infancy. Only a small proportion of molluscan biodiversity has been explored, and most available evidence remains in vitro. Therefore, low yields, high purification costs, limited bioavailability, potential allergenicity, processing and storage instability, and regulatory uncertainty do not preclude low-volume niche applications, but they continue to hinder cost-effective scale-up and broader commercialization in mass-market food and nutraceutical products. Future studies should move beyond peptide identification and focus on integrated strategies for product development. These should include optimized enzymatic hydrolysis, bioinformatics-guided peptide screening, molecular docking, improved purification approaches, encapsulation-based delivery systems, and rigorous safety and efficacy evaluation.

## Figures and Tables

**Table 1:** Preparation strategies and characteristics of mollusk peptides.

Preparation Strategy	Mollusk Protein Source	Key Processing Parameters	Representative Peptide Characteristics	Functional Effects	Ref.
Enzymatic hydrolysis	Entire soft tissue of *Crassostrea rivularis*	0.05% Alcalase (*w*/*w* protein foundation); pH 8.0; 50 °C; 8 h of hydrolysis	Mostly low-molecular-weight peptides (<1 kDa); high soluble nitrogen concentration; enriched. In hydrophobic amino acids and metal-chelating residues	Antioxidant and aphrodisiac effects in male mice	[33]
Enzymatic hydrolysis	Freeze-dried meat powder of *Crassostrea talienwhanensis*	Papain (pH 6.5, 55 °C, 3 h), Neutrase (pH 7.0, 50 °C, 3 h), Alcalase (pH 9.8, 50 °C, 3 h), enzyme dose 2500 U per gram of substrate	Dominated by low-molecular-weight peptides (<1 kDa, 87–97%), rich in basic and hydrophobic amino acids	In vitro antioxidant activity, including DPPH radical scavenging and reducing power; the <1 kDa fractions showed the greatest antioxidant benefits	[34]
Enzymatic hydrolysis	Entire soft tissue of commercially produced live mollusk	Pepsin hydrolysis; pH 1.8; 40 °C; 5 h; enzyme-substrate ratio 1.5:100 (*w*/*w*, protein basis)	Mostly coordinated via amino nitrogen and carboxylate oxygen atoms, zinc-binding peptide is enriched in His, Glu, Asp, Lys, Arg	Enhanced zinc-binding capacity and gastrointestinal stability	[35]
Microbial fermentation	Homogenized whole soft tissue	Water-mollusk ratio 3:1 (*w*/*v*); honey 6% (*w*/*v*) as carbon source; pH 4.5; inoculated with Saccharomyces	Fermentation broth enriched in protein-derived peptides and free amino acids	Increased sperm quality, testosterone levels, antioxidant enzyme activity (CAT, SOD, and GSH-PX), and reduced lipid peroxidation	[36]
Ultrasound-assisted hydrolysis	Total soluble protein extracted from fresh meat	Mollusk protein to water ratio of 1:7 (*m*:*v*); ultrasound pretreatment at 20 kHz, 200 W for 20 min (2 s on/2 s off, in an ice bath); sequential hydrolysis using pepsin (5000 U⋅g^−1^ protein, 37 °C, 2 h) and trypsin (5000 U⋅g^−1^ protein, 37 °C, 2 h)	Two bioactive peptides, DSQLAPFRF and HFNPRL, were identified among 248 sequences. These peptides are mostly made of 9–18 amino acids and showed high predicted bioactivity and strong binding affinity to bone morphogenetic protein-2	Reduced H_2_O_2_-induced oxidative stress in MC3T3-E1 cells, promoting osteoblast proliferation, differentiation, and mineralization	[37]
Ultrasound-assisted hydrolysis	Fresh mollusk homogenate	The homogenate was generated using a 1:2 meat/water ratio, ultrasound pretreatment at 20 kHz with 450 W for 20 min, and enzymatic hydrolysis with Neutrase-Flavourzyme (1:1, *w*/*w*) at 1:2 enzyme/substrate ratio, pH ~7.0, 50 °C, 4 h	Increased amino acid nitrogen content; enrichment of umami- related amino acids (Glu, Asp) and short peptide fragments contributing to taste and reactivity	Promoted peptide liberation; facilitated downstream functional and sensory enhancement	[38]
Heat-assisted hydrolysis	Isolate from *Magallana gigas*	Heat for 15 min at 100 °C; in vitro digestion with pepsin (pH 2, 37 °C, 2 h) and trypsin (pH 8, 37 °C, 4 h); 5:1 enzyme/protein (U mg 1)	Peptides enriched in hydrophobic residues; six novel ACE inhibitory peptides identified	In vitro ACE inhibitory activity; altered peptide profiles and digestibility	[39]

**Table 2:** Sequence-resolved mollusk-derived peptides reported in the recent literature (2016–2026).

Biological/Pharmacological Activity	Peptide Sequence(s)	Taxonomic Group	Source Species/Material	Specific Function/Activity	Preparation Route	Main Use Direction	Evidence Level	Ref.
Hypocholesterolemia	APDMAFPR; QDAVMPNPF; LPAYPMP; DYPRPW	Gastropod	*Abalone viscera*, *Haliotis discus hannai*	HMGCR inhibition; reduced cholesterol micelle solubility and TC/TG in hyperlipidemic HepG2 cells.	Enzymatic hydrolysis, ultrafiltration (<1 kDa), LC-MS/MS identification, docking and in vitro validation	Food/nutraceutical/pharmacy lead	Cellular (HepG2)	[46]
Antioxidant	DEDEDEDK	Gastropod	*Abalone*, *Haliotis discus hannai*	Antioxidant; interferes with Keap1-Nrf2 interaction and reduces AAPH-induced oxidative stress in HUVECs.	In silico peptide-library screening from virtual enzymatic hydrolysis	Food/nutraceutical	Cellular (HUVECs)	[12]
Anticancer	KVEPQDPSEW (AATP)	Gastropod	*Abalone*, *Haliotis discus hannai*	Anticancer/anti-metastatic; suppresses vasculogenic mimicry and migration/invasion in HT1080 cells.	Isolated peptide	Pharmacy/anticancer lead	Cellular (HT1080 cells)	[47]
Neuromodulatory	alpha-conotoxin AtIA/At1.1: ZGCCSHPACSVNHPELCW (Z = pyroglutamate)	Gastropod	*Cone snail*, *Conus ateralbus*	Neuromodulatory; inhibits alpha7 nicotinic acetylcholine-receptor-associated calcium responses in DRG-neuron assay.	Endogenous venom-derived conopeptide	Pharmacy/neuropharmacology	Cellular (DRG neurons)	[48]
Analgesic	Ziconotide/omega-conotoxin MVIIA: CKGKGAKCSRLMYDCCTGSCRSGKC	Gastropod	*Cone snail*, *Conus magus*	FDA/EMA-approved N-type voltage-gated calcium-channel blocker for severe refractory chronic pain; intrathecal analgesic.	Synthetic version of an endogenous venom conopeptide originally isolated from Conus magus	Pharmacy/approved analgesic	Human (clinical trials)	[49]
Antihypertensive	IVTNWDDMEK; VGPAGPRG	Gastropod	*Deep-sea snail*, *Volutharpa ampullacea perryi*	ACE-inhibitory/antihypertensive-related activity; review table reports endothelial-cell protection context.	Edible-part protein hydrolysate	Food/nutraceutical/antihypertensive lead	Cellular (endothelial cells)	[50]
Antimicrobial	Nv-p1: SGRGKGGKGLGKGGAKRHR; Nv-p2: SGRGKGGKGLGKGGAKRH; Nv-p3: KKKPTKK	Gastropod	*Marine snail*, *Nerita versicolor*	Antimicrobial and antibiofilm activity.	Endogenous peptide pool; nanoLC-ESI-MS/MS, database de novo identification	Pharmacy/antimicrobial lead	In vitro	[45]
Anti-inflammatory	ALGTWK	Gastropod	*Ventral harp*/*harp snail*, *Harpa ventricosa visceral mass*	Anti-inflammatory; reported to downregulate NO, TNF-alpha and IL-1beta in LPS-stimulated RAW264.7 macrophages.	Visceral-mass-derived peptide	Pharmacy/anti-inflammatory lead	Cellular (RAW264.7 macrophages)	[51]
Antihypertensive	FRVW; LPYY	Bivalvia	*Akoya pearl oyster flesh*, *Pinctada fucata*	ACE inhibitory activity.	Alcalase hydrolysis of pearl-oyster meat	Food/pharmacy lead	In vitro (ACE assay)	[50]
Antihypertensive	GVGSPY	Bivalvia	*Akoya pearl oyster shell*, *Pinctada fucata*	ACE inhibitory activity.	enzymatic hydrolysis	Food/pharmacy lead	In vitro (ACE assay)	[50]
Antihypertensive	EVMAGNLYPG	Bivalvia	*Blue mussel*, *Mytilus edulis*	Antihypertensive activity reported in spontaneously hypertensive rats.	Fermentation-derived peptide	Food/pharmacy lead	Animal (spontaneously hypertensive rats)	[50]
Antihypertensive	MFR; MFV; FV; KP; QP; QVK; IK; YKV; IRK; MLKV; NFRPQ; YEGDP; WF; GPE; SWISS; SVEWK; FKWH	Bivalvia	*Blue mussel*, *Mytilus edulis*	ACE inhibitory; IK, YEGDP, WF and SWISS showed strong activity; YEGDP/WF protected HUVECs from oxidative injury.	Enzymatic protein hydrolysate	Food/nutraceutical	Cellular (HUVECs; plus in vitro ACE assay)	[52]
Antioxidant	YPPAK	Bivalvia	*Blue mussel*, *Mytilus edulis*	Antioxidant; DPPH, hydroxyl-radical and superoxide-radical scavenging; anti-lipid-peroxidation activity.	Neutrase hydrolysate	Food/preservation/nutraceutical	In vitro (radical-scavenging assays)	[53]
Antihypertensive	VW; LGW; MVWT	Bivalvia	*Blue mussel*, *Mytilus edulis muscle*	ACE-inhibitory/antihypertensive activity.	Alcalase hydrolysis of mussel muscle	Food/dietary supplement/antihypertensive lead	In vitro (ACE assay)	[50]
Antihypertensive	WPMGF	Bivalvia	*Clam*, *Cyclina sinensis*	ACE inhibitory; stable under simulated gastrointestinal digestion.	Trypsin hydrolysis, ultrafiltration	Functional food/dietary supplement	In vitro (ACE assay and simulated digestion)	[16]
Antihypertensive	TAY; VK; KY; FYN; YA	Bivalvia	Cross-linked oyster proteins	Acute antihypertensive activity in spontaneously hypertensive rats.	Enzymatic hydrolysis of cross-linked oyster protein	Food/pharmacy lead	Animal (spontaneously hypertensive rats)	[50]
Anti-inflammatory	EGLLGDVF	Bivalvia	*Green mussel*, *Perna viridis foot*	Anti-inflammatory; stable and active in LPS-stimulated RAW264.7 macrophages.	Peptide isolated from green mussel foot protein fraction/hydrolysate	Food/pharmacy lead	Cellular (RAW264.7 macrophages)	[54]
Antihypertensive	VISDEDGVTH	Bivalvia	*Japanese littleneck clam*, *Ruditapes philippinarum*	Antihypertensive-related activity; reported to promote nitric-oxide production and improve renal vascular context in animal model.	Bacillus natto fermentation-derived clam peptide	Food/pharmacy lead	Animal (vascular/renal model)	[50]
Antidiabetic	KLNSTTEKLEE; TDDAHKF	Bivalvia	*Noble scallop adductor muscle*, *Chlamys nobilis*	Alpha-glucosidase inhibitory activity with antioxidant relevance; reported IC_50_ values of 144.89 and 136.96 micromolar.	Computational screening, enzymatic hydrolysate LC-MS/MS identification	Food/antidiabetic lead	In vitro (enzyme IC_50_)	[55]
Antihypertensive	AEYLCEAC	Bivalvia	*Oyster*, *Crassostrea gigas*	ACE inhibitory; competitive inhibition with antihypertensive effect in spontaneously hypertensive rats.	Simulated gastrointestinal digestion of oyster hydrolysate	Food/nutraceutical/antihypertensive lead	Animal (hypertensive rats)	[56]
Antihypertensive	DLTDY; DY	Bivalvia	*Oyster*, *Crassostrea gigas*	Antihypertensive activity in spontaneously hypertensive rats after oral administration/dietary exposure.	Trypsin hydrolysis of oyster tissue	Food/pharmacy lead	Animal (spontaneously hypertensive rats)	[50]
Antihypertensive	HLHT; GWA	Bivalvia	*Pearl oyster flesh*, *Pinctada fucata martensii*	Antihypertensive activity after intravenous administration in rat model.	Alkaline-protease hydrolysis of pearl-oyster meat	Pharmacy/antihypertensive lead	Animal (rat model)	[50]
Antihypertensive	AGFAGDDAPR; CDVDIR; IIAPPER; IWHHTFYNGLR; GIQTAVR	Bivalvia	*Scallop skirt*, *Chlamys farreri*	Antihypertensive activity in spontaneously hypertensive rats.	Bacillus natto fermentation of scallop skirt	Food/nutraceutical	Animal (spontaneously hypertensive rats)	[50]
Antimicrobial	Octominin: GWLIRGAIHAGKAIHGLIHRRRH	Cephalopoda	*Long-arm octopus*, *Octopus minor*	Anticandidal/antifungal activity; damages fungal cells and shows antibiofilm relevance.	Synthetic peptide derived from an Octopus minor defense-protein sequence	Pharmacy/antifungal lead	Animal (zebrafish infection model)	[57]
Antioxidant	AQNY; AMMLAW; FEGAW; GGAW; VDTVVCVW; VVCLW	Cephalopoda	Octopus protein hydrolysate	Antioxidant; GGAW showed the strongest activity and protected IEC-6 cells against H_2_O_2_-induced oxidative stress.	Alcalase hydrolysis	Food/nutraceutical	Cellular (IEC-6 cells)	[58]
Metabolic (ACE/DPP-IV/PEP inhibitory)	SHPDLRLA; LGMP (+15.99) GRL; GNK (+15.99) GPVGPL; additional ACE-active fractions include EGRGLP (+15.99) GL and LGRGEP (+15.99) GL	Cephalopoda	Squid-processing by-products	ACE, DPP-IV and PEP inhibitory activities depending on hydrolysate fraction.	Processing-derived enzymatic hydrolysis with Esperase/Alcalase/Trypsin	Food/metabolic-health ingredient/by-product valorization	In vitro (ACE, DPP-IV, and PEP assays)	[59]

**Table 3:** PTM effects in mollusk-derived peptides.

Structural Feature or Modification	Main Molecular Effect	Functions Most Affected	Representative Mollusk Peptide Context
Disulfide bonds and connectivity	Fixes 3D scaffold, controls folding, and presents pharmacophore residues	Receptor selectivity, antimicrobial activity, stability	Conotoxins, myticins, mussel defensins
Gamma-carboxyglutamate (Gla)	Enables divalent cation binding and metal-dependent conformational switching	NMDA receptor modulation and structural stabilization	Conantokin-G/T and Gla-containing conopeptides
D-amino acids	Increases protease resistance and creates noncanonical binding geometry	Stability, receptor selectivity, neuromodulatory activity	Contryphan and D-amino-acid-containing conopeptides
C-terminal amidation	Neutralizes terminal charge and protects against carboxypeptidases	Receptor binding, ion-channel activity, stability	Many mature conopeptides, including conotoxin drug leads
N-terminal pyroglutamate or cyclization	Blocks aminopeptidase attack and restricts terminal flexibility	Stability and receptor-binding persistence	Modified conopeptides and synthetic analogs
Bromotryptophan	Increases indole size, hydrophobicity, and polarizability	Receptor interaction and peptide chemical diversity	BrTrp-containing Conus peptides
O-glycosylation	Increases hydrophilicity, solubility, and protease shielding	Stability, distribution, receptor interaction	Contulakin-G and glycosylated conopeptides
Tyrosine sulfation	Introduces strong negative charge and modifies electrostatic recognition	Receptor or ion-channel interaction	Sulfated alpha-conotoxins

## Data Availability

No new data were created or analyzed in this study. Data sharing is not applicable to this article.

## Supplementary Materials

The following supporting information can be downloaded at: https://www.mdpi.com/article/10.3390/molecules31183186/s1. Table S1: Search concepts and representative terms; Table S2: Amino acid composition versus reported antioxidant potency of mollusk-derived peptides; Table S3: Indicative regulatory pathways for mollusk-derived peptides according to bioactivity and region.
