# Supplementary material for: Mollusk-Derived Peptides at the Food-Pharma Interface: Bioactivities, Preclinical Evidence, and Development Prospects
Source: Molecules. 2026 Sep 10;31(18):3186. doi: 10.3390/molecules31183186 (PMC13609028; doi:10.3390/molecules31183186)
Supplement: Supplementary file 1 [file molecules-31-03186-s001.zip › Table S1.pdf]

Supplementary 1

Literature search method and criteria

This article was conducted as a narrative review, rather than a systematic review or meta-analysis, to integrate evidence on mollusk-derived bioactive peptides across food, nutraceutical, and pharmaceutical contexts. PubMed/MEDLINE, Web of Science Core Collection, Scopus, and Google Scholar were searched from database inception to August 2026. Search strings combined four concept blocks covering molluscan taxa and source materials, peptide classes, production and characterization methods, and biological activities or applications (Table S1). Terms within each block were combined with OR, the four blocks were combined with AND, and syntax was adapted for each database. Reference lists of eligible primary studies and pertinent reviews were also searched manually. The principal evidence window was 2020 to 2026 because the review emphasizes contemporary enzymatic and processing strategies, mass-spectrometry-based sequence identification, mechanism-oriented validation, and recent translational applications. Earlier publications were retained selectively when they reported clinical milestone, established a widely used analytical or production method.

Studies were included when they reported at least one relevant feature: species or tissue source; enzymatic hydrolysis, fermentation, extraction, fractionation, or purification; peptide sequence, structure, mass-spectrometric identification, or post-translational modification; biological or pharmacological activity and mechanism; stability, bioavailability, safety, or allergenicity; or food, nutraceutical, pharmaceutical, delivery, packaging, or cosmeceutical application. Food-derived hydrolysates, endogenous immune and adhesive peptides, venom-derived conopeptides, and synthetic analogues based on molluscan sequences were eligible. Non-molluscan marine peptide or collagen studies were retained only when they provided clearly identified contextual evidence for processing or application and were not treated as direct evidence of mollusk-derived efficacy. Records focused solely on non-peptide metabolites, unrelated organisms, editorials, conference abstracts, non-peer-reviewed material, duplicates, or reports lacking sufficient methodological or biological detail were excluded. Computational-only predictions could inform the discussion of discovery approaches but were not included as sequence-resolved efficacy evidence without experimental validation; unresolved hydrolysate mixtures were discussed narratively but were not entered as individual peptide sequences. Owing to heterogeneity in peptide composition, assays, dose metrics, comparators, and outcomes, findings were synthesized narratively; no quantitative pooling or formal risk-of-bias scoring was performed.

Table S1. Search concepts and representative terms.

Concept	Representative search terms
Mollusk source	Mollusk or mollusc or shellfish or gastropod or bivalve or cephalopod or oyster or mussel or clam or scallop or Abalone or snail or cone snail or Conus or octopus or squid or cuttlefish
Peptide class	Peptide or bioactive peptide or protein hydrolysate or collagen peptide or antimicrobial peptide or immune peptide or adhesive peptide or conopeptide or conotoxin or venom peptide
Production and characterization	Enzymatic hydrolysis or simulated gastrointestinal digestion or fermentation or extraction or ultrafiltration or chromatography or RP-HPLC OR LC-MS/MS or mass spectrometry or sequence identification or proteomic or transcriptomic or venomic or molecular docking or post-translational modification
Activity and application	Antioxidant or ACE inhibitory or antihypertensive or anti-inflammatory or antimicrobial or antiviral or anticancer or antidiabetic or DPP-IV inhibitory or mineral binding or mineral chelation or anti-fatigue or immunomodulatory or gut health or microbiome or wound healing or nutraceutical or functional food or pharmaceutical or drug development or food packaging or cosmeceutical
