# Supplementary material for: Mollusk-Derived Peptides at the Food-Pharma Interface: Bioactivities, Preclinical Evidence, and Development Prospects
Source: Molecules. 2026 Sep 10;31(18):3186. doi: 10.3390/molecules31183186 (PMC13609028; doi:10.3390/molecules31183186)
Supplement: Supplementary file 1 [file molecules-31-03186-s001.zip › Table S2.pdf]

Table S2 Amino acid composition versus reported antioxidant potency of mollusk-derived peptides

Species / material	Peptide	Y	W	H	P	Hydrophob. (%)	Reported potency / endpoint	Reference
Abalone (Haliotis discus hannai)	DEDEDEDK	0	0	0	0	0.0	No peptide IC_{50} reported; 1000 µg/mL reduced AAPH-induced ROS	[1]
Oyster (Crassostrea rivularis)	AWVDY	1	1	0	0	80.0	IC_{50}: DPPH 27.50 ± 4.55; hydroxyl 44.00 ± 6.04; superoxide 18.00 ± 3.08 µg/mL	[2]
Oyster (Crassostrea rivularis)	MSFRFY	1	0	0	0	66.7	IC_{50}: DPPH 50.75 ± 7.12; hydroxyl 89.75 ± 22.85; superoxide 16.50 ± 1.52 µg/mL	[2]
Oyster (Crassostrea rivularis)	EPLRY	1	0	0	1	40.0	IC_{50}: DPPH 28.25 ± 5.35; hydroxyl 36.50 ± 7.79; superoxide 16.50 ± 3.84 µg/mL	[2]
Oyster (Crassostrea rivularis)	RKPPWPP	0	1	0	4	14.3	IC_{50}: DPPH 166.75 ± 8.52; hydroxyl 303.75 ± 16.06; superoxide 84.00 ± 5.42 µg/mL	[2]
Oyster (Crassostrea rivularis)	YAKRCFR	1	0	0	0	42.9	IC_{50}: DPPH 21.75 ± 7.08; hydroxyl 18.75 ± 6.49; superoxide 11.00 ± 2.65 µg/mL	[2]
Blue mussel (Mytilus edulis)	YPPAK	1	0	0	2	40.0	EC_{50}: DPPH 2.62; hydroxyl 0.228; superoxide 0.072 mg/mL	[3]
Octopus protein hydrolysate	AQNY	1	0	0	0	50.0	No peptide-specific IC_{50} reported	[4]
Octopus protein hydrolysate	AMMLAW	0	1	0	0	100.0	No peptide-specific IC_{50} reported	[4]
Octopus protein hydrolysate	FEGAW	0	1	0	0	60.0	No peptide-specific IC_{50} reported	[4]
Octopus protein hydrolysate	GGAW	0	1	0	0	50.0	No peptide-specific IC_{50} reported; strongest among the six by ORAC/ABTS	[4]
Octopus protein hydrolysate	VDTVVCVW	0	1	0	0	62.5	No peptide-specific IC_{50} reported	[4]
Octopus protein hydrolysate	VVCLW	0	1	0	0	80.0	No peptide-specific IC_{50} reported	[4]
Jumbo squid (Dosidicus gigas) skin gelatin	FDSGPAGVL	0	0	0	1	44.4	Hydroxyl-radical IC_{50} 90.90 µM	[5]
Jumbo squid (Dosidicus gigas) skin gelatin	NGPLQAGQPGER	0	0	0	2	16.7	Hydroxyl-radical IC_{50} 100.72 µM	[5]

References

1. Wu, J., et al., *Screening and Antioxidant Activities Evaluation of Peptides From Abalone (Haliotis discus hannai Ino).* Food Science & Nutrition, 2025. **13**(2): p. e70028.
2. Huang, H., et al., *Isolation and characterization of antioxidant peptides from oyster (Crassostrea rivularis) protein enzymatic hydrolysates.* Food Science & Nutrition, 2023. **11**(1): p. 261–273.
3. Verma, A.K., et al., *Peptides From Animal and Fishery Byproducts: Uplifting the Functionality of Fifth Quarter.* Food Science & Nutrition, 2025. **13**(4): p. e70140.
4. Peng, B., B. Cai, and J. Pan, *Octopus-derived antioxidant peptide protects against hydrogen peroxide-induced oxidative stress in IEC-6 cells.* Food Science & Nutrition, 2022. **10**(11): p. 4049–4058.
5. Alemán, A., et al., *Contribution of Leu and Hyp residues to antioxidant and ACE-inhibitory activities of peptide sequences isolated from squid gelatin hydrolysate.* Food Chemistry, 2011. **125**(2): p. 334–341.
