# Supplementary material for: Mollusk-Derived Peptides at the Food-Pharma Interface: Bioactivities, Preclinical Evidence, and Development Prospects
Source: Molecules. 2026 Sep 10;31(18):3186. doi: 10.3390/molecules31183186 (PMC13609028; doi:10.3390/molecules31183186)
Supplement: Supplementary file 1 [file molecules-31-03186-s001.zip › Table S3.pdf]

Table S3 Indicative regulatory pathways for mollusk-derived peptides according to bioactivity and region

Bioactivity / proposed positioning	United States (FDA)	European Union (EFSA / European Commission)	China (NHC / SAMR / NMPA)
**ACE inhibition / antihypertensive activity**	Likely food or dietary-supplement route only for a non-disease structure/function claim (e.g., supporting maintenance of blood pressure already within the normal range), with substantiation. A novel supplement ingredient may require an NDI notification.<br>Conventional-food use may require GRAS or food-additive status. Claims to lower or treat hypertension place the product in the drug pathway.	Food/food-supplement route is possible only with an authorized health claim and compliance with its conditions of use.<br>A peptide-specific claim would require EFSA assessment and European Commission authorization.<br>A novel hydrolysate or isolated peptide may also require Novel Food authorization. Claims to treat or prevent hypertension indicate a medicinal product.	The 2023 health-food catalogue includes 'helps maintain blood pressure at a healthy level.' A health-food registration/filing route and claim-specific evidence would be required;<br>A novel peptide ingredient may first require NHC new-food-ingredient review. Claims to treat hypertension indicate the NMPA drug pathway.
**Anti-inflammatory activity**	A carefully framed claim such as supporting a healthy inflammatory response may qualify as a dietary-supplement structure/function claim, depending on its full context and substantiation.<br>Claims to reduce inflammation associated with arthritis, inflammatory bowel disease, or another disease indicate a drug. Ingredient-level NDI/GRAS requirements may still apply.	A food or supplement cannot carry a peptide-specific anti-inflammatory health claim unless that claim is authorized after scientific assessment.<br>A novel ingredient may require Novel Food authorization. Presentation for treating or preventing inflammatory disease indicates a medicinal product.	Anti-inflammatory' is not itself one of the 24 functions in the 2023 health-food catalogue. A food route would require a legally permitted alternative function that is scientifically applicable and supported;<br>An unlisted function should not be assumed. Disease-treatment or prevention claims indicate the NMPA drug pathway.
**Antioxidant activity**	A substantiated dietary-supplement structure/function claim such as supporting antioxidant defenses or helping maintain cell integrity may be possible.<br>Claims that the peptide prevents cancer, cardiovascular disease, or another disease are not structure/function claims and require an authorized health-claim or drug pathway, as applicable. NDI/GRAS requirements may apply.	Only an authorized health claim may be used for a food or food supplement, subject to the authorized wording and conditions. Peptide-specific antioxidant wording would require EFSA assessment and European Commission authorization.<br>Novel Food review may also be required. Disease prevention or treatment indicates the medicinal pathway.	The 2023 health-food catalogue includes 'helps antioxidant activity.' Products using this claim require the applicable health-food registration/filing route and supporting evidence; novel peptide ingredients may require NHC review.<br>Disease prevention or treatment claims are prohibited for foods and indicate a drug pathway.
**Anticancer activity**	'Anticancer,' 'prevents cancer,' and 'treats cancer' are disease claims and place the product in the drug pathway.<br>A separate general antioxidant structure/function claim cannot be used to imply anticancer efficacy.	Presentation for preventing or treating cancer indicates a medicinal product. A food health-claim route cannot substitute for the evidence and authorization required for anticancer therapy;<br>disease-risk-reduction food claim would require separate specific authorization.	Cancer prevention and treatment claims are not permitted for ordinary foods or health foods.<br>A mollusk peptide developed with an anticancer indication would require the NMPA drug-development and marketing-authorization pathway.
**Mineral-binding / mineral-bioavailability activity**	A conventional food or dietary supplement may use a substantiated non-disease structure/function claim concerning normal mineral absorption or bone support.<br>A new ingredient may require NDI notification or GRAS/food-additive clearance<br>Claims to prevent or treat osteoporosis or anemia require an authorized disease-risk-reduction claim or drug pathway, as applicable.	A food/supplement route is possible only through an authorized health claim whose wording and conditions of use fit the ingredient and mineral. A novel peptide chelate or hydrolysate may require<br>Novel Food authorization.<br>Treatment or prevention of disease indicates a medicinal product.	Relevant permitted health-food functions include 'helps improve bone density' and 'improves iron-deficiency anemia,' depending on the product and evidence.<br>Health-food registration/filing is required, and a novel ingredient may require NHC review.<br>Claims beyond an authorized function may indicate the drug route.
**Anti-fatigue activity**	Claims supporting normal energy, endurance, or recovery from occasional fatigue may be dietary-supplement structure/function claims if substantiated and not linked to a disease.<br>Claims to treat pathological fatigue or a disease-associated symptom indicate a drug. NDI/GRAS requirements may apply.	A food or supplement may use only an authorized health claim under its conditions of use; peptide-specific anti-fatigue wording would require assessment and authorization.<br>Novel Food review may apply.<br>Treatment of pathological fatigue indicates a medicinal product.	The 2023 health-food catalogue includes 'relieves physical fatigue.' The applicable health-food registration/filing route and supporting testing are required;<br>a novel ingredient may require NHC review.<br>Disease-related fatigue claims indicate the drug pathway.

Bioactivity / proposed positioning	United States (FDA)	European Union (EFSA / European Commission)	China (NHC / SAMR / NMPA)
**Glycemic-control / antihyperglycemic activity**	A claim supporting normal glucose metabolism or maintaining blood glucose already within the normal range may be a dietary-supplement structure/function claim if substantiated.<br>Claims to lower elevated glucose or treat/prevent diabetes indicate a drug.<br>Ingredient-level NDI/GRAS requirements may apply.	A food/supplement claim concerning glucose control must be specifically authorized and used under its conditions.<br>Novel Food authorization may also be required.<br>Claims to prevent or treat diabetes indicate a medicinal product.	The 2023 health-food catalogue includes 'helps maintain blood glucose at a healthy level.' Health-food registration/filing and claim-specific evidence are required;<br>a novel peptide ingredient may require NHC review. Diabetes-treatment or prevention claims indicate the NMPA drug pathway.
**Body-fat / lipid-metabolism activity**	Substantiated claims supporting normal lipid metabolism or body composition may fit a dietary-supplement structure/function route if they avoid obesity, hyperlipidemia, or cardiovascular-disease treatment claims.<br>NDI/GRAS requirements may apply.<br>Disease-treatment claims indicate a drug.	Food or supplement claims relating to body weight or lipids require prior authorization and compliance with the authorized wording and conditions.<br>Novel Food review may apply.<br>Treatment of obesity, dyslipidemia, or cardiovascular disease indicates a medicinal product.	The 2023 catalogue includes 'helps control body fat' and 'helps maintain blood lipids (cholesterol/triglycerides) at healthy levels.' Health-food registration/filing and supporting evidence are required;<br>novel ingredients may require NHC review. Disease-treatment claims indicate the NMPA drug route.
**Gut-health / microbiome-interacting activity**	Claims supporting normal digestive function or a healthy gut microbiome may qualify as dietary-supplement structure/function claims if substantiated.<br>Claims to prevent infection or treat inflammatory bowel disease, ulcers, or other gastrointestinal disease indicate a drug.<br>NDI/GRAS requirements may apply.	A microbiome or digestive-health claim for a food/supplement must be authorized and used under its conditions;<br>demonstrating a compositional microbiome change alone may not establish a beneficial physiological effect.<br>Novel Food review may apply.<br>Disease-treatment claims indicate a medicinal product.	Relevant permitted health-food functions include 'helps regulate intestinal flora,' 'helps digestion,' 'helps bowel movement,' and 'provides auxiliary protection of the gastric mucosa.' Health-food registration/filing is required;<br>new ingredients may require NHC review. Disease claims indicate the NMPA drug pathway.

Abbreviations and agency roles. NDI, new dietary ingredient; GRAS, generally recognized as safe; NHC, National Health Commission; SAMR, State Administration for Market Regulation; NMPA, National Medical Products Administration. In the EU, EFSA performs scientific assessments, while the European Commission and Member States make authorization decisions. In China, NHC reviews new food ingredients, SAMR administers health-food registration/filing and permitted functions, and NMPA administers drugs.
